# B7-H3 in Cancer Immunotherapy—Prospects and Challenges: A Review of the Literature

**DOI:** 10.3390/cells14151209

**Published:** 2025-08-06

**Authors:** Sylwia Mielcarska, Anna Kot, Miriam Dawidowicz, Agnieszka Kula, Piotr Sobków, Daria Kłaczka, Dariusz Waniczek, Elżbieta Świętochowska

**Affiliations:** 1 Department of Medical and Molecular Biology, Faculty of Medical Sciences in Zabrze, Medical University of Silesia in Katowice, 19 Jordana St., 41-800 Zabrze, Poland; s85876@365.sum.edu.pl (A.K.); s86196@365.sum.edu.pl (P.S.); s73707@365.sum.edu.pl (D.K.); 2 Department of Oncological Surgery, Faculty of Medical Sciences in Zabrze, Medical University of Silesia, 40-514 Katowice, Poland; d201069@365.sum.edu.pl (M.D.); d201070@365.sum.edu.pl (A.K.); dwaniczek@sum.edu.pl (D.W.)

**Keywords:** B7-H3 (CD276), immune checkpoint, solid tumors, B7-H3 clinical trials

## Abstract

In today’s oncology, immunotherapy arises as a potent complement for conventional cancer treatment, allowing for obtaining better patient outcomes. B7-H3 (CD276) is a member of the B7 protein family, which emerged as an attractive target for the treatment of various tumors. The molecule modulates anti-cancer immune responses, acting through diverse signaling pathways and cell populations. It has been implicated in the pathogenesis of numerous malignancies, including melanoma, gliomas, lung cancer, gynecological cancers, renal cancer, gastrointestinal tumors, and others, fostering the immunosuppressive environment and marking worse prognosis for the patients. B7-H3 targeting therapies, such as monoclonal antibodies, antibody–drug conjugates, and CAR T-cells, present promising results in preclinical studies and are the subject of ongoing clinical trials. CAR-T therapies against B7-H3 have demonstrated utility in malignancies such as melanoma, glioblastoma, prostate cancer, and RCC. Moreover, ADCs targeting B7-H3 exerted cytotoxic effects on glioblastoma, neuroblastoma cells, prostate cancer, and craniopharyngioma models. B7-H3-targeting also delivers promising results in combined therapies, enhancing the response to other immune checkpoint inhibitors and giving hope for the development of approaches with minimized adverse effects. However, the strategies of B7-H3 blocking deliver substantial challenges, such as poorly understood molecular mechanisms behind B7-H3 protumor properties or therapy toxicity. In this review, we discuss B7-H3’s role in modulating immune responses, its significance for various malignancies, and clinical trials evaluating anti-B7-H3 immunotherapeutic strategies, focusing on the clinical potential of the molecule.

## 1. Introduction

Cancer is one of the leading causes of death worldwide [1]. In recent decades, the possibilities of cancer treatment have increased in number, with targeted therapies and immunotherapies emerging as potent complements to conventional strategies [2]. Immunotherapeutic strategies, aiming to boost anti-cancer immunity, are based upon the fact that tumor cells develop multiple protective mechanisms to escape host immune responses, which are reinforced in the process of immune editing, minimizing cancer cell immunogenicity. The immune tolerance induced by tumors abrogates the proper recognition of altered antigens on cancer cells due to the expression of various ligands that bind to inhibitory receptors on immune cells, suppressing their functions. Hence, the idea of “immune checkpoint” blockade (ICB) emerged as one solution for the interruption of immune-inhibitory pathways induced by cancer cells [3]. Nowadays, ICB, including anti-CTLA-4 antibodies or anti-PD1/PD-L1 strategies, is successfully utilized in the treatment of several malignancies. Still, large groups of patients exhibit resistance to therapies involving immune checkpoint inhibitors (ICIs). In consequence, searching for novel immune checkpoints with therapeutic potential is crucial [2].

B7/CD28 family proteins play an essential role in modulating immune responses, providing co-inhibitory and co-stimulatory signals that maintain immunological homeostasis. In the event of tumorigenesis, mechanisms involving these molecules may contribute to establishing an immunosuppressive microenvironment, enabling cancer immune evasion [4,5]. Among the known members of the B7 family, the clinical potentials of PD-1/PD-L1 (B7-H1, CD274), PD-L2 (B7-DC, CD273), and VISTA (B7-H5) have been studied the most extensively [5,6]. B7-H3 (CD276), initially described as a co-stimulatory molecule, has been identified by numerous authors as a potent suppressor of anti-tumor responses [7,8]. In humans, the molecule appears in two isoforms, 2 IgB7-H3 (B7-H3 VC) and 4 IgB7-H3 (B7-H3 VCVC), and its gene is located on chromosome 15 [8,9]. Moreover, a soluble form of B7-H3 (sB7-H3) has been discovered in human serum and extracellular vesicles [10]. Although B7-H3 levels in healthy tissues are relatively low, the molecule is abundantly expressed in numerous cancer types, such as lung cancer, esophageal squamous cell carcinoma (ESCC), gastric cancer, pancreatic cancer, colorectal cancer, liver cancer, breast cancer, brain tumors, and prostate cancer [11]. Furthermore, its upregulation can boost cancer cell proliferation and metastatic abilities, or mediate drug resistance, consequently leading to poorer patient outcomes [12,13]. In this review, we focus on the significance of B7-H3 in tumorigenesis in various malignancies, its influence on tumor-associated immunity, and recent therapeutic strategies involving this molecule. However, the vast subjects of B7-H3 receptors and molecular pathways, as well as B7-H3’s role in gastrointestinal tumors, reach beyond this manuscript and have been discussed in our previous review [14].

## 2. B7-H3 in Tumorigenesis

### 2.1. B7-H3 in the Regulation of Immune Responses

#### 2.1.1. The Influence of B7-H3 on Immune Cell Infiltrations in Tumors

The role of B7-H3 in modulating immunity in tumorigenesis and tumor progression has been studied extensively. The protein has been detected across various cancer types, prominently expressed on diverse immune cell populations, including macrophages, monocytes, dendritic cells (DCs), neutrophils, and myeloid-derived suppressor cells (MDSCs). In addition to its presence on immune cells, B7-H3 expression has also been observed on cancer cells, in the tumor-associated vasculature, and carcinoma-associated fibroblasts (CAFs) [15,16]. Initial reports described B7-H3 as a costimulatory molecule with the potential of boosting anti-tumor T-cell function. Early studies have posited that the interaction between B7-H3 and the triggering receptor expressed on myeloid cells (TREM)-like transcript 2 (TLT-2, TREML2) significantly contributes to augmented production of IFN-γ and enhanced CD8 T-cell cytotoxic activity in mice [17]. Furthermore, anti-B7-H3 monoclonal antibody (mAb) treatment has been shown to promote tumor growth in parental squamous cell carcinoma SCCVII cell line models (*p* = 0.0005), while adenoviral transfer of B7-H3 resulted in significant tumor size reduction in mouse models of colon cancer [18,19]. Notably, CD8 T cells appear to selectively eliminate B7-H3-expressing target cells, resulting in diminished tumorigenicity. On the other hand, Leitner et al. reached a contradictory conclusion, providing no evidence for the B7-H3-TLT-2 interaction in human T-cells. Instead, they characterized the molecule as a potential inhibitor of T-cell activity [20]. Despite the increasing number of studies, the precise mechanisms underlying B7-H3's modulation of immune responses and its role in anti-tumor activity remain inadequately understood, warranting further investigation into its unidentified receptor.

In the context of tumor-infiltrating lymphocytes (TILs) and cytokine regulation, research is increasingly emphasizing the co-inhibitory role of B7-H3 and its associated pro-tumoral effects. Still, the relationship between B7-H3 and immune infiltration patterns remains contentious. Elevated B7-H3 has been implicated in immune-cold tumors [21,22]. Immune-cold tumors, characterized by a worse response to immune checkpoint blockade (ICB) therapies [23], feature a lowered density of tumor-infiltrating lymphocytes (TILs), elevated immunosuppressive properties of tumor microenvironment (TME), and, therefore, compromised capabilities of inducing anti-cancer immunological responses [24]. In contrast, immune-hot tumors, with strong immune infiltrations, are believed to demonstrate increased potential for ICB therapy responsiveness [24]. In various malignancies, B7-H3 was negatively correlated with CD8+ T-cell frequencies [25]. Loss of B7-H3 could augment the number of intratumor CD8+ and CD4+ T-cells; however, its influence on the CD4 TILs remains vague [21,26]. Interestingly, Cattaneo and colleagues found a positive association between cytoplasmic B7-H3 and CD8+ T-cell numbers in PDAC samples [27]. In addition, in non-small cell lung carcinoma (NSCLC), B7-H3 expression was positively correlated with CD45+ immune cells, with increased expression corresponding to heightened numbers of immune cells (*p* = 0.002), CD8+ T cells (*p* = 0.011), and plasmacytoid dendritic cells (*p* = 0.015) [16]. These results highlight the complexity of the B7-H3 influence on immune cells, which may reach beyond the regulation of cell density.

#### 2.1.2. Relationship Between B7-H3 and Cytokine Secretion

Importantly, the regulatory functions of B7-H3 concern the expression of a vast range of cytokines, thus modulating both pro-inflammatory and anti-inflammatory effects. For instance, B7-H3 has been shown to inhibit T-cell proliferation mediated by antigen-presenting cells (APCs) or antibody binding. Its silencing resulted in a substantial increase in interferon-γ (IFN-γ) levels within murine models of airway inflammation under Th1 conditions [28]. In ovarian cancer, there was a negative correlation between B7-H3 expression and the presence of TNF-α+IFN-γ+ CD8+ T cells. CD8+ tumor-infiltrating lymphocytes (TILs) from B7-H3-deficient mice demonstrated increased expression of the degranulation marker CD107a and proliferation marker Ki67, alongside elevated production of IFN-γ, TNF-α, and granzyme B [29]. These observations suggest that tumor-induced B7-H3 upregulation leads to T-cell exhaustion. In addition, B7-H3 has been proposed to influence T-cell cytotoxicity through the interaction with 4-1BB, a tumor necrosis factor (TNF) receptor on TILs. The blockade of this receptor boosted the cytotoxic function of CD8 T-cells, while B7-H3 knockdown augmented IFN-γ and TNF-α levels in serum in xenograft models [30]. Different results were obtained by Meng et al., who showed that B7-H3 enhanced TNF-α secretion, while the cytokine could upregulate the expression of B7-H3 in CRC [31]. In addition, B7-H3 expression was enhanced through IL-6 and STAT3 signaling in mice [32]. These reports highlight complex signaling pathways that could be involved in immune evasion in CRC. Research by Leitner et al. [20] demonstrated that B7-H3 can impede human T-cell proliferation and functionality in the presence of activation signals. The molecule was shown to suppress IL-2 production, with T-cell activity restored by exogenous IL-2 administration. Additionally, B7-H3 was found to diminish the production of notable cytokines, including IFN-γ, IL-2, IL-10, and IL-13, in both CD4 and CD8 T cells, with these effects applicable to both B7-H3 isoforms, 4 Ig B7-H3 and 2 Ig B7-H3-Ig. Evidence suggests that B7-H3 contributes to the inhibition of CD8 T-cell anti-tumor activity across multiple malignancies, with some research implicating its role in regulating CD4 T-cell functions [29,32]. Liu and colleagues posited that mTORC1 induces the phosphorylation of the transcription factor Yin-Yang 2 (YY2), consequently leading to B7-H3 upregulation and suppression of CD4 T-cell activity. Notably, CD4+ T cells appear particularly essential for the anti-tumor effects observed following B7-H3 inhibition in mouse models [26]. Furthermore, B7-H3 has been shown to negatively regulate Th1 responses, with its expression in dendritic cells being notably enhanced by interferon-γ (a Th1-associated cytokine) and diminished by interleukin-4 (IL-4), a Th2 cytokine [28]. Interestingly, B7-H3 was also considered essential for the secretion of both Th1 and Th2 cytokines within the TME in another study, suggesting a complex regulatory landscape. Th1 cells are well documented for their ability to mediate anti-tumor responses through the destruction of malignant cells [33]. Conversely, Th2 lymphocytes may antagonize Th1 functions, often fostering a pro-tumor milieu while also having roles in mitigating cancer progression, highlighting their dualistic nature in the TME [34]. Further investigation into the specific influence of B7-H3 on Th1 and Th2 lymphocyte dynamics is warranted.

Moreover, B7-H3 expression could be induced by Tumor Growth Factor-β (TGF-β)—an anti-inflammatory cytokine—promoting proliferation during carcinogenesis and implicated in cancer cell invasiveness [25]. Through pathways involving SMAD3/4, miR-155, the transcription factor CEBPB, and miR-143, TGF-β1 upregulation led to increased expression of B7-H3 and B7-H4 in CRC cells [35]. Importantly, Zhou et al. demonstrated the existence of a negative feedback loop in the autocrine secretion mechanisms of colorectal cancer as the overexpression of B7-H3 reduced the production of IL-4, IL-6, IL-17, TGF-β1, and TNF-α in HCT-116 cells. At the same time, B7-H3 levels influenced T-cell-dependent secretion within the tumor microenvironment (TME): B7-H3 upregulation increased IL-2, IL-6, IL-17, and TGF-β1 levels while diminishing IFN-γ production [35]. Altogether, B7-H3 differently regulates the secretion within cancer cells and TME. Such observations may explain contradictory results of studies investigating the relationship between the molecule and cytokine expression. Furthermore, the elements of tumor microenvironment are not only modulated by B7-H3 but also take an active part in the regulation of B7-H3 expression, highlighting the complex web of interactions between the molecule and TME.

#### 2.1.3. B7-H3 and Regulatory T Cells

The relationship between B7-H3 expression in tumors and the presence of regulatory T cells (Tregs) remains ambiguous. As a CD4+ cell subset that exerts immunosuppressive functions, Tregs play a significant role in maintaining immune tolerance [36]. Research conducted by Inamura et al. indicated a positive correlation between B7-H3 expression levels and the density of FOXP3+ Tregs in clear cell renal cell carcinoma (ccRCC) [15]. Notably, high B7-H3 expression—both in tumor cells and vasculature—was associated with poor prognosis in FOXP3+ high groups, contrarily showing no significant prognostic impact in FOXP3-low samples. These findings suggest that B7-H3 might facilitate pro-tumor effects via FOXP3+ Tregs within the TME of ccRCC [15]. In another study, the expression of B7-H3 mRNA was found to be significantly correlated with the density of regulatory FOXP3+ T cells, whereas no such correlation was observed at the protein level [37]. Additionally, a distinct subpopulation of carcinoma-associated fibroblasts (CAFs) identified in breast cancer, termed CAF-S1, exhibited elevated expression of B7-H3 and demonstrated immunosuppressive properties. This specific CAFs population was capable of inhibiting T-cell proliferation and, through mechanisms involving B7-H3, promoting the differentiation of T cells into a CD25+FOXP3+ regulatory phenotype [38]. However, the results concerning the associations between B7-H3 expression and regulatory T cells are contradictory, including some authors demonstrating none, or a negative correlation with the mentioned regulatory populations [27,29,39]. These findings imply that B7-H3 may play an important role in the differentiation and activity of regulatory cells, contributing to immune escape in certain malignancies, but the significance of such function is likely dependent on cancer type.

#### 2.1.4. Macrophage Function

Several studies have indicated that elevated expression of B7-H3 facilitates M2 macrophage functions. M2 macrophages foster immunosuppressive activities that can contribute to tumorigenesis, while M1 macrophages play a more significant role in promoting anti-tumor responses [40]. Notably, B7-H3 expression has been positively correlated with levels of M2 macrophage markers, such as CD68 and CD163. Experimental silencing of B7-H3 in tumor cells within murine models resulted in a significant reduction in the population of M2 macrophages [22]. Consistently, B7-H3 treatment was shown to enhance the expression of M2 macrophage markers while concurrently reducing levels of TNF-α and HLA-DR, which are characteristic of M1 macrophages [41]. Furthermore, a significant positive correlation between B7-H3 and CCL2 levels has been observed; targeting B7-H3 resulted in decreased M2 macrophage populations as well as inhibited their recruitment through the downregulation of CXCL1 and CCL2 within the tumor microenvironment (TME) [22]. These findings indicate that B7-H3 may enhance the differentiation and migration of M2 macrophages through the CCL2–CCR2 axis, suggesting a potential role for B7-H3 in promoting an immunosuppressive tumor microenvironment (TME) by supporting M2 macrophage dominance. However, Miller et al. revealed that elevated B7-H3 expression correlated with higher numbers of pro-inflammatory M1 macrophages in their analysis of 156,791 samples [25]. Further investigation by Zhang et al. revealed that B7-H3 enhances the anti-apoptotic capacities of macrophages in lung cancer, where silencing B7-H3 led to heightened macrophage apoptosis under hypoxic conditions. The authors suggested that these effects are mediated through B7-H3-dependent upregulation of HIF-1α via NF-κB activation. B7-H3 was also found to be upregulated on monocytes/macrophages within the TME, leading to increased infiltration of these cells into tumors. It was proposed that the expression of CD276 on macrophages may be regulated by miR-29a-3p delivered by exosomes. Inhibition of exosome secretion resulted in increased intracellular levels of miR-29a-3p, subsequently downregulating B7-H3 mRNA. Conversely, THP-1-derived macrophages decreased intracellular miR-29a-3p levels and elevated B7-H3 expression through exosome transport, which contributed to apoptosis inhibition and cancer progression [42]. Future research endeavors are essential to elucidate the mechanisms underpinning B7-H3-mediated apoptosis in macrophages, its regulatory pathways, and the influence of CD276 on macrophage behavior and cancer phenotypes. The precise function of B7-H3 in modulating macrophage differentiation, activity, and polarization remains to be clarified and may vary according to the specific cancer type.

#### 2.1.5. B7-H3 in the Regulation of NK Cell Function

B7-H3 has been shown to significantly modulate natural killer (NK) cells functionality, which is crucial for anti-tumor immunity via direct cytotoxicity and cytokine production [43]. In various malignancies, a notable positive correlation exists between B7-H3 expression and NK cell infiltrates; however, this molecule was found to inhibit NK cell cytotoxicity. This inhibition is regulated by the Myc oncogene, which downregulates the expression of miR-29c—a microRNA that targets B7-H3—resulting in B7-H3 overexpression and consequent impairment of NK cell cytotoxic activity. Furthermore, B7-H3 has been observed to activate Myc through phosphorylation, creating a feedback loop [44]. In neuroblastoma (NB), Pathania et al. documented the downregulation of miR-29a, miR-29b, and miR-29c, noting a negative correlation with B7-H3 expression. These specific miRNAs have the capacity to target B7-H3 mRNA within NB cells, promoting its degradation and facilitating augmented NK cell activation and cytotoxic response [45]. This mechanism may partially elucidate the role of B7-H3 in promoting immune evasion in cancer. On the other hand, Xiong and colleagues suggested a different regulatory mechanism. In mice with induced esophageal squamous cell carcinoma (ESCC), B7-H3 overexpression fostered the formation of neutrophil extracellular traps (NETs) via CXCL1–CXCR2 signaling activation. NETs could impair the function of NK cells, leading to reduced NK cell numbers and facilitated tumorigenesis. Consequently, B7-H3 silencing decreased CXCL1 levels, and inhibited NET formation, thus increasing NK cell infiltrations [46]. Moreover, the elevated expression of B7-H3 in neutrophils was associated with a poorer prognosis of gastric cancer patients [46]. These results show that B7-H3 may impair NK cell functions through various pathways, contributing to the immune evasion of cancer cells.

#### 2.1.6. B7-H3 and Other Immune Cell Subsets

The expression of B7-H3 was also positively correlated with CD11b and CD33, myeloid-derived suppressor cell (MDSC) markers, in head and neck squamous cell carcinoma [39]. MDSCs form an immune cell subset with immunosuppressive properties contributing to cancer progression [47]. Notably, B7-H3 knockout resulted in an 80% reduction of the CD11b+Ly6G+ polymorphonuclear MDSC population, indicating that MDSCs may serve as a vital component in B7-H3-mediated immune evasion in mouse models [26]. Collectively, these findings underscore the multifaceted role of B7-H3 in various signaling pathways that contribute to the intricate regulation of the immune milieu in malignancies. Future investigations aimed at elucidating the specific receptors, ligands, and pathways through which B7-H3 exerts its immunomodulatory effects are essential for the development of targeted and effective therapeutic strategies.

In conclusion, the molecule impacts various immunoregulatory mechanisms during cancer development and progression, fostering immunosuppressive responses and immune evasion in different types of malignancies. Still, the pathways of B7-H3s’ influence are poorly understood. Further investigations concerning the role of the molecule in immunity are necessary for the development of effective therapeutic strategies. The influence of B7-H3 on immune microenvironment in tumors discussed is presented in Table 1.

### 2.2. B7-H3 and Its Non-Immune Functions in Tumorigenesis

Additionally, the molecule may facilitate tumorigenesis through various pathways independently of its role in immunity. In breast cancer, B7-H3 has been shown to activate the MEK signaling pathway through its interactions with major vault protein (MVP), leading to the proliferation of cancer stem cells in vitro [63]. Furthermore, B7-H3 induced epithelial–mesenchymal transition (EMT), thereby promoting lung metastasis and contributing to a more aggressive disease phenotype via the Raf/MEK/ERK pathway [63]. Similar pro-tumorigenic effects have been documented in gastric and ovarian cancers, where B7-H3 modulated the Jak2/STAT3 signaling pathway [64,65]. Moreover, B7-H3 influenced EMT processes in glioblastoma, melanoma, and hepatocellular carcinoma by upregulating matrix metalloproteinases MMP-2 and MMP-9 or downregulating E-cadherin levels [66,67,68].

B7-H3’s role in the invasion and migration of cancer cells may also be mediated via signaling pathways such as PI3K/AKT and p38/ERK MAPK, as demonstrated in Clear Cell Renal Cell Carcinoma (CRCC) and Non-Small Cell Lung Cancer (NSCLC) [69,70].

Interestingly, Sutton et al. demonstrated that B7-H3 facilitates tumor-intrinsic proliferation of cancer cells, with B7-H3 knockout leading to a 50–80% reduction in the growth of the cervical cancer cell line when compared to wild-type (WT) cells. Additionally, murine models with B7-H3 knockdown exhibited diminished tumor growth rates and significantly improved overall survival in contrast to those with B7-H3-expressing tumors. Notably, the application of 4 Ig-B7-H3 dimers, detectable in live cells, exerted even more profound proliferative effects, correlating with enhanced activation of key signaling pathways, including AKT, Jak/STAT, HIF1α, and NF-κβ, relative to 4 Ig-B7-H3 alone [71].

Furthermore, Meng et al. highlighted B7-H3's involvement in lysophosphatidic acid (LPA) signaling. In lung cancer fibroblasts, LPA was shown to upregulate B7-H3 expression via the LPAR1 receptor. This interaction subsequently enhanced both fibroblast and non-small cell lung cancer (NSCLC) cell proliferation through the LPAR1/B7-H3 signaling axis. Given that the inhibition of both B7-H3 and LPAR1 could impede CAFs' proliferation and subsequently reduce NSCLC cell proliferation and tumor growth, it would be prudent to explore LPAR1/B7-H3 inhibitory strategies in NSCLC treatment regimens [72].

B7-H3 is also an important modulator of glucose metabolism. In neuroblastoma (NB), B7-H3 has been identified as a crucial regulator of 6-phosphofructo-2-kinase/fructose-2,6-bisphosphatase 3 (PFKFB3) expression, enhancing glucose uptake and promoting lactic acid production. Notably, B7-H3 knockdown reduced levels of c-Met and phosphofructokinase platelet (PFKP) while increasing phosphorylated Stat3. Preclinical studies using xenograft mouse models demonstrated that B7-H3 silencing inhibited tumor growth, indicating that B7-H3 may exacerbate the severity of NB by modulating the Stat3/c-Met pathway and augmenting glucose turnover in cancer cells [73], findings that align with recent studies by Wu et al. in lung cancer. In this study, B7-H3 increased cell proliferation and migration through PI3K/AKT signaling, which it did by modulating α-enolase (ENO1) activity. ENO1, while physiologically involved in glycolysis, also promotes an aggressive cancer phenotype by directly interacting with B7-H3. While B7-H3 does not directly influence ENO1 expression, it enhances ENO1's enzymatic activity [73].

Moreover, Yang et al. found that B7-H3 expression positively correlated with the levels of vessel biomarkers CD34 and VEGF in the synovial tissue of rheumatoid arthritis (RA) patients, as well as in collagen-induced arthritis (CIA) mice. Silencing B7-H3 in CIA mice resulted in decreased CD34 expression, potentially inhibiting B7-H3-dependent angiogenesis, although the mechanisms by which B7-H3 regulates angiogenesis in RA remain unclear [74]. These findings may help understand the role of the molecule in neoangiogenesis in tumors.

The results discussed above prove that B7-H3 exerts its pro-tumor functions on numerous levels, including the modulation of EMT, cell metabolism, and angiogenesis, creating complex regulatory associations between tumor cells and the elements of TME. Exploring them in more detail could be a goal for future studies tackling the issue of B7-H3 in tumorigenesis. The non-immune functions of the molecule in the context of gastrointestinal tumors were described more broadly in our previous review [14].

## 3. B7-H3 in Tumors

### 3.1. The Role and Clinical Potential of B7-H3 in Melanoma

The upregulation of B7-H3 expression in melanoma has been substantiated through analyses of multiple publicly available datasets, including the Cancer Genome Atlas (TCGA), GEPIA, and the Human Protein Atlas. A variety of observational cohort studies utilizing immunohistochemistry (IHC) have confirmed elevated protein expression levels of B7-H3 in both primary melanoma lesions and metastases [75]. High B7-H3 expression has been linked to enhanced migration and invasion capabilities of melanoma cells through the upregulation of MMP2 and modulation of the STAT3 pathway [67]. B7-H3 mRNA expression was significantly elevated in advanced stages of disease and positively correlated with shortened DSS [76]. In the immune context, high levels of B7-H3 were associated with reduced infiltration of tumor-infiltrating lymphocytes and an increase in tumor fibrosis, characteristics that typify the armored-cold phenotype of melanoma—an expression profile associated with low immune checkpoints expression and arising from this resistance to conventional anti-PD-1/PD-L1 agents [75]. In the mouse model of melanoma, the blockade of B7-H3 expression augmented the cytotoxicity of CD8+ T cells and NK cells. Additionally, B7-H3 has been shown to reduce the efficacy of dacarbazine (DTIC) chemotherapy, along with small-molecule inhibitors targeting the MAP kinase (MAPK) and AKT/mTOR pathways in vitro; conversely, inhibition of B7-H3 enhanced the responsiveness of metastatic melanoma cells to these therapeutic agents [77]. Given the established positive correlation between high B7-H3 expression and the prometastatic potential of melanoma cells, various strategies aimed at targeting B7-H3 have been proposed. Zhang et al. demonstrated the efficacy of chimeric antigen receptor T-cell (CAR T) therapy targeting B7-H3 in combating melanoma cells [78]. Huang et al. developed a bispecific T-cell engaging (BiTE) antibody-based mRNA therapy, encapsulating mRNA encoding B7-H3 × CD3 BiTE within ionizable lipid nanoparticles (LNPs). Application of this BiTE mRNA-LNP in a mouse model resulted in robust BiTE protein expression and a notable antitumor response against melanoma, marked by a reduction in metastatic lesions [79]. Ventin et al. have developed a B7-H3 CAR T-cell therapy incorporating an inducible caspase-9 (iCas9) suicide gene, which effectively damages uveal melanoma cells in vitro and reduces liver metastases in a mouse model [80]. Presently, several anti-B7-H3 agents are undergoing clinical evaluation, including enoblituzumab (an anti-B7-H3 monoclonal antibody), CAR T-cell immunotherapy, and an antibody–drug conjugate (ADC) comprising an anti-B7-H3 monoclonal antibody linked to a synthetic duocarmycin analogue (Vobramitamab duocarmazine). Melanoma patients with tumors expressing B7-H3 are enrolled in a phase 1 study assessing the safety profile of enoblituzumab in conjunction with pembrolizumab [81].

### 3.2. Brain Tumors—A Potent Direction for Anti-B7-H3 Therapies

B7-H3 has been reported to be widely expressed in several brain tumors such as glioblastoma (GBM), diffuse intrinsic pontine glioma (DIPG), neuroblastoma, medulloblastoma, atypical teratoid/rhabdoid tumor, craniopharyngioma, and brain metastases [60,61,62,67,82,83,84,85,86]. In recent years, its therapeutic potential in these malignancies has been studied extensively. The role of CD276 in brain tumors has been recently reviewed [86].

#### 3.2.1. Gliomas

B7-H3 was overexpressed in glioma, showing higher mRNA expression than other B7 family members [60]. Its levels were increased in CSF, blood serum, and tumors of high-grade glioma patients compared to low-grade glioma [87]. Additionally, Shen and colleagues uncovered that among three B7-H3 isoforms (2, 3, and 4 Ig), 4 Ig is commonly expressed in glioma, and 2 Ig is mainly present in highly malignant tumors, while 3 Ig barely occurs in the disease. Interestingly, RNA binding protein annexin A2 (ANXA2) regulated both B7-H3 isoforms, but RNA methyltransferase NOP2/Sun RNA methyltransferase 2 (NSUN2) or 5-methylcytosine reader Y-box binding protein 1 (YBX1) enabled the production of 2 Ig [88]. Elevated molecule expression in CSF or tumor tissue was associated with higher grade of the disease, IDH mutation status, molecular subtype, dysfunctional T-cell phenotype, and worse survival [87,89]. Importantly, B7-H3 has been shown to increase MMP-2/-9 and decrease E-cadherin expression to promote EMT. CD276 also induced glioma cell proliferation and invasion via upregulation of the JAK2/STAT3/Slug pathway [66]. Moreover, B7-H3 was involved in pathways related to T-cell receptor signaling and angiogenesis [89].

#### 3.2.2. Glioblastoma

In contrast to a recent study [88], 4 Ig B7-H3 isoform was detected in glioblastoma (GBM) by Digregorio et al., while 2 Ig was present in non-cancerous brain tissue. The authors, therefore, suggested 4 IgB7-H3 as a potential therapeutic target [90]. In GBM, B7-H3 was found to be associated with poor differentiation, high expression of glioma self-renewing cell (GSC)-related genes, and Myc expression. B7-H3 inhibition led to the downregulation of Myc expression and a decrease in tumor growth in a human GBM cell line xenograft mouse model [91]. Wang G. et al. developed oncolytic adenovirus (oAds) with CXCL11 to reverse the immunosuppressive tumor microenvironment and enhance infiltration of CAR T-cells targeting B7-H3 in glioblastoma. Application of modified adenovirus improved antitumor immunity by increasing the proportion of CD8+ cells, NK cells, and M1 polarized macrophages, which are powerful effectors in antitumor response, with a simultaneous decrease in the proportion of immunosuppressive cell populations such as MDSCs, Tregs, and M2-polarized macrophages [92]. Modified B7-H3-targeted CAR T-cells with an incorporated cytoplasmatic unit of IL7Ralpha showed enhanced and prolonged antitumoral effects against GBM in vitro and in vivo [93]. In another study, B7-H3-targeted CAR-T was enhanced by the administration of interleukin-7-loaded oncolytic adenovirus (oAD-IL7) to provide additional activating signal for T cells [94]. Currently, one clinical trial of CAR T-cell therapy to B7-H3 (NCT04077866) is in progress to assess the safety and efficacy profile in patients with refractory or recurrent GBM (phase I/II study, status recruiting) [95]. Also, other therapeutic modalities targeting B7-H3 are developed in GM. B7-H3-targeted ADC with conjugated auristatin E showed remarkable toxicity against glioblastoma cells in vitro and in a murine model with a selective accumulation of drug within the intratumoral space [96]. B7-H3 antibody labeled 131I decreased the proliferation of GB cells and induced transformation of tumor phenotype from “cold” to “hot” by increasing number of CD4+ and CD8+ infiltrating tumors and inducing polarization of M2 macrophages to M1 in mouse models [97].

#### 3.2.3. Diffuse Intrinsic Pontine Glioma (DIPG)

Diffuse intrinsic pontine glioma (DIPG) is a pediatric brain tumor associated with poor prognosis and a short median survival [98]. DIPG shows upregulation in B7-H3, as revealed by proteomics analysis [99]. Additionally, in a study on nine DIPG patients, all specimens had B7-H3 immunoreactivity, showing moderate or strong CD276 staining intensity, but the molecule was present in more than 50% of cells only in one case. No correlation with OS was found [83]. The correlations between CD276 and clinicopathological characteristics of patients or TME have not been evaluated; therefore, the issue could be addressed in future reports. In a study conducted by Vitanza et al., B7-H3 CAR T-cell-treated mice showed improved survival, and a first-in-human phase I trial, BrainChild-03 (NCT04185038), demonstrated that DIPG patients tolerated well the repeated locoregional administering of B7-H3 CAR T cells. Thus, future studies involving larger numbers of patients could focus on such therapeutic strategies [100].

#### 3.2.4. Neuroblastoma

Neuroblastoma (NB) is an aggressive embryonal malignancy of the sympathetic nervous system, rarely located in the brain [101]. B7-H3 is upregulated in the tumors, while its high levels correspond to a more advanced tumor stage and poorer survival [45,102]. Tian et al. found that B7-H3 was highly expressed in NB and was coexpressed with glypican 2 (GPC2) in 95% of NB samples [103]. Still, data regarding the associations between B7-H3 and clinicopathological charackteristics of patients or prognosis is limited.

The influence of CD276 on NB progression is also poorly defined. Zhu et al. found that B7-H3 expression in NB cells promoted their proliferation, migration, and invasion, while its silencing inhibited these effects. Moreover, augmented expression of the molecule increased glucose metabolism through the Stat3/c-Met pathway [73]. According to another study, increased B7-H3 degradation by miR-29 family elevated NK cell activation in NB, decreased macrophage infiltration and microvessel density, and induced apoptosis of tumor cells in vitro and in patient-derived xenograft tumors. This means that B7-H3 could shape the immune landscape in NB, inhibiting anti-tumor activity. The authors suggested that augmenting miRNA levels to target CD276 could be another potential therapeutic option that could be explored in future studies [45]. Moreover, Xiang and colleagues demonstrated that lncRNA NUTM2A-AS1 inhibited B7-H3 degradation, leading to cisplatin resistance in NB in vitro [104]. Such results confirm that B7-H3-targeting could be used in combination with other therapeutic strategies to enhance their effectiveness.

Recently, several anti-B7-H3 approaches have been implied for NB treatment. Tian and colleagues suggested that combined targeting of CD276/GPC2 by CAR T-cell therapies could serve as an effective treatment strategy, decreasing the risk of immune evasion. Moreover, CAR T cells targeting both CD276 and GPC2 killed NB cells with high efficacy even when one of these molecules was absent [103]. In another study, Disialoganglioside 2 (GD2)—combined with B7-H3-targeting with bispecific antibodies—resulted in improved therapy selectivity in GD2-positive NB models [105]. Additionally, the B7-H3-targeting antibody–drug conjugate (ADC) m276-SL-PBD, showing a strong anti-tumor activity, has been proposed for NB treatment in pediatric patients [106]. Another ADC against the B7-H3 antigen—Vobramitamab duocarmazine (MGC018)—showed cytotoxicity against all CD276-positive NB cell lines analyzed but did not destroy NB cells with no B7-H3 expression. In mouse models, the treatment could delay tumor growth and prolong mouse survival [107]. In another study, Kramer et al. demonstrated in their phase I study that compartmental radioimmunotherapy (cRIT) combined with the anti-B7-H3 murine monoclonal antibody omburtamab could be a promising strategy for NB treatment [108]. Thus, CD276 seems to be a potent therapeutic target for NB therapy. Nevertheless, its role in the disease should be characterized in more detail.

#### 3.2.5. Medulloblastoma

Medulloblastoma (MB) is the most prevalent malignant brain tumor, occurring predominantly in childhood [109]. Li et al. detected B7-H3 expression in 96% of medulloblastoma cases. Moreover, 71% of malignancies exhibited CD276 expression in more than 25% of tumor cells. Furthermore, tumors with high B7-H3 expression demonstrated reduced infiltration of γδ T lymphocytes and CD3+ T lymphocytes [61]. Elevated CD276 was also linked to poorer survival outcomes in another study [110].

Interesting results regarding the role of CD276 in MB were obtained by Purvis et al. B7-H3 upregulation in MB cells increased exosomal secretion, suggesting that B7-H3 may play a role in exosome production. Such exosomes could then influence cancer and stromal cells, shaping the microenvironment in MB. The exosomes derived from B7-H3-overexpressing (OE) cells contained increased fractions of molecules associated with chemokine and cytokine signaling pathways, molecules related to JAK/STAT, angiogenesis, PDGF, PI3K, and glycolysis-associated signaling pathways. The presented mechanism may thus illustrate a novel way for CD276 to induce angiogenesis, metastasis, and cancer progression in MB [111].

Several therapeutic strategies focused on CD276 have been proposed for MB treatment. Mews and colleagues introduced multivalent bispecific chemically self-assembling nanorings (CSANs) that targeted CD3ε and B7-H3. αB7-H3-αCD3 CSANs effectively augmented TIL numbers and induced the lysis of MB cells. CSANs could cross the blood-tumor barrier with high efficacy, and the authors suggested that they could be used as a novel therapeutic strategy to reduce non-specific T-cell activation in MB treatment [112]. Recently, Shishido et al. showed that the enhancer of zeste homolog 2 (EZH2) targeting reduced B7-H3 expression in MB and decreased cancer cell viability. According to the authors, such a therapy could be combined with strategies targeting B7-H3 [113]. However, the specific role of B7-H3 in medulloblastoma remains blurry, and more research on the topic could help in developing successful therapies.

#### 3.2.6. Craniopharyngioma

A craniopharyngioma (CP) is an uncommon epithelial tumor, located in the sellar and parasellar region [114,115]. Recently, Coy et al. showed that B7-H3 was highly expressed in papillary craniopharyngioma [116]. Additionally, in a study conducted by Wang and colleagues, all 132 CP samples were positive for B7-H3 [117]. Moreover, high CD276 levels correlated with worse survival, poorer T-cell infiltrations, and elevated IBA1^+^ macrophages, meaning that the molecule may shape the immune landscape in CP, leading to a more aggressive course of the disease. BiTE antibodies targeting B7-H3 could effectively inhibit CP cell proliferation and induce their lysis [62]. In addition, Tang et al. established a CP organoid model, revealing that ADC exhibited robust tumor suppression in 3D models. These findings suggest that targeting B7-H3 with antibody–drug conjugates may be a promising therapeutic strategy for craniopharyngioma treatment [118]. Despite the clinical potential of B7-H3 targeting in CP, little is known about its role in the disease.

### 3.3. B7-H3 in Lung Cancer—Clinical Utility and Options for Combined Therapy

#### 3.3.1. Non-Small Cell Lung Cancer (NSCLC)

In non-small cell lung cancer (NSCLC), which accounts for a substantial majority of lung cancer cases, the prevalence of tumors exhibiting upregulated B7-H3 expression is reported to range from 32% to 70%, depending on the study cohort and the methodologies employed for expression assessment [119,120]. Notably, a comprehensive study by Mehmet A. et al. involving a large cohort of 634 patients revealed that an impressive 80.4% of cases demonstrated positive B7-H3 staining [121]. Additionally, elevated levels of B7-H3 were detected in both serum and pleural effusions of NSCLC patients, with serum concentrations significantly higher in NSCLC compared to individuals with other nonmalignant pulmonary conditions and healthy controls. Furthermore, serum levels in pleural effusions were found to correlate with TNM staging, highlighting the potential utility of B7-H3 as a biomarker for NSCLC [122]. The high expression of B7-H3 has been associated with poorer histological differentiation, lymph node involvement, and advanced clinical staging [123]. In in vitro studies, it has been demonstrated that circular RNA hsa_circ_0000896 plays a role in the upregulation of B7-H3 in NSCLC by degrading miR-15a-5p, a microRNA known to silence B7-H3 expression in normal tissues [124]. Inhibiting B7-H3 expression in NSCLC cell lines has resulted in a marked decrease in the migratory and invasive capabilities of cancer cells, indicating that B7-H3 may facilitate NSCLC progression not only through immune-related mechanisms but also via the upregulation of proteins associated with integrins [125]. In NCLC, B7-H3 is coexpressed with various immune checkpoints, revealing distinct coexpression patterns across different subpopulations of immune cells. Interestingly, the coexpression rates of B7-H3 with PD-L1 and B7H4 are notably low, suggesting that tumors may preferentially utilize a singular pathway for immune evasion [121]. The prognostic significance of B7-H3 expression in NSCLC remains controversial, with several studies indicating a positive correlation between high B7-H3 levels and poor survival outcomes, while other investigations have not corroborated this association. A meta-analysis conducted by Wu S. et al., encompassing a total of 864 NSCLC patients, suggested a lack of significant impact of B7-H3 expression on overall survival rates, highlighting the need for further research to elucidate the relationship between B7-H3 and patient prognosis in NSCLC [120].

#### 3.3.2. Small-Cell Lung Cancer (SCLC)

Small-cell lung cancer (SCLC) represents 13–15% of all lung cancer cases. This highly aggressive neuroendocrine carcinoma is associated with a poor prognosis and a low five-year survival rate [126]. The use of Immune Checkpoint Inhibitors (ICIs) in treating SCLC is currently approved only for first-line therapy in advanced disease, often in combination with conventional chemotherapy. However, the efficacy of this approach is limited by a high risk of treatment resistance, and recurrence, and consequently a modest impact on survival rates [127]. Therefore, exploring and identifying new potential therapeutic targets is essential to fully harness the potential of immunotherapy for SCLC treatment. Two studies have shown that over 60% of SCLC tumors are positive for B7-H3 staining, with B7-H3 expression occurring significantly more frequently than PD-L1 expression, which ranges from 7% to 13% in SCLC cases [127,128]. B7-H3 levels were also found to correlate with tumor size and reduced overall survival [127]. High expression of B7-H3 was detected in a new SCLC cell line developed from malignant pleural effusion, making it the third most expressed antigen, indicating the potential for selective targeting of SCLC cells [129]. To date, B7-H3 is being extensively evaluated in two clinical trials that are assessing the utility of B7-H3 inhibitors in the treatment of SCLC [130].

### 3.4. Breast Cancer—B7-H3 as a Potential Prognostic Marker and Therapeutic Target

Numerous studies have demonstrated the overexpression of B7-H3 across various histological types of breast cancer [50,131]. The expression rate of B7-H3 exhibits significant variability among cancer subtypes and study cohorts, ranging from 73.4% in ductal carcinoma in situ (DCIS) tumors to 90.6% in cohorts predominantly comprising invasive ductal and lobular carcinomas [132]. Furthermore, it has been established that B7-H3 levels increase during the transformation of phyllodes tumors into malignant neoplasms [133]. Therefore, the soluble form of B7-H3 present in serum has been proposed as a potential biomarker for breast cancer, capable of distinguishing patients with malignant lesions from both healthy individuals and those with benign breast lesions; however, the study evaluating the diagnostic potential of B7-H3 was limited by small study sample size [134]. Due to the high upregulation of B7-H3 in cancer tissues compared to very low expression levels in normal tissues, there has been growing interest in leveraging B7-H3 for targeted imaging of breast cancer, particularly through the application of microbubbles conjugated to B7-H3-targeted antibodies [135].

B7-H3 in breast cancer is implicated in mechanisms of immune evasion, tumor progression, and resistance to therapeutic interventions. B7-H3 overexpression was related to reduced infiltration of CD3+ and CD8+ T cells [133]. Associations with the involvement of lymph nodes and more advanced stages of the disease, which was confirmed by meta-analyses [136]. High B7-H3 levels were found in breast cancer brain metastases, suggesting significant involvement of this immune checkpoint in promoting cancer aggressiveness [137]. In breast cancer cell lines, B7-H3 diminished the proliferative capacity of CD4+ and CD8+ T cells and downregulated IFN-γ production through the modulation of mTOR signaling [138]. In triple-negative breast cancer (TNBC), B7-H3 was upregulated in tumor-associated macrophages and fostered tumor progression by creating an immunosuppressive tumor microenvironment, which subsequently promoted tumor angiogenesis and inhibited T-cell infiltration [139]. The coexpression pattern characterized by high B7-H3 and low PD-L1 expression, along with poor lymphocyte infiltration, suggests an “immune cold” phenotype in TNBC tumors expressing B7-H3 [140]. Research by Huang et al. highlighted the role of aberrant glycosylation in B7-H3 stabilization in TNBC, protecting the molecule from ubiquitination [141]. In mouse models of TNBC, the inhibition of B7-H3 via monoclonal antibodies has been shown to convert immune-cold tumors into immune-hot tumors, thereby enhancing the effectiveness of anti-PD-L1 therapy [140]. Similarly, Interventions aimed at inhibiting abnormal B7-H3 glycosylation improved responses to anti-PD-L1 therapy [141]. What is more, silencing B7-H3 expression has been found to enhance the sensitivity of cancer cells to mTOR inhibitors and paclitaxel by disrupting the JAK2/STAT3 signaling pathway [140,142], while B7-H3-induced activation of the Raf/MEK/ERK signaling has been implicated in promoting lung metastasis [143].

B7-H3 in breast cancer is implicated in mechanisms of immune evasion, tumor progression, and resistance to therapeutic interventions. B7-H3 overexpression was related to reduced infiltration of CD3+ and CD8+ T cells [133]. Associations with the involvement of lymph nodes and more advanced stages of the disease, which was confirmed by meta-analyses [136]. High B7-H3 levels were found in breast cancer brain metastases, suggesting significant involvement of this immune checkpoint in promoting cancer aggressiveness [137]. In breast cancer cell lines, B7-H3 diminished the proliferative capacity of CD4+ and CD8+ T cells and downregulated IFN-γ production through the modulation of mTOR signaling [138]. In triple-negative breast cancer (TNBC), B7-H3 was upregulated in tumor-associated macrophages and fostered tumor progression by creating an immunosuppressive tumor microenvironment, which subsequently promoted tumor angiogenesis and inhibited T-cell infiltration [139]. The coexpression pattern characterized by high B7-H3 and low PD-L1 expression, along with poor lymphocyte infiltration, suggests an “immune coldȁ phenotype in TNBC tumors expressing B7-H3 [140]. Research by Huang et al. highlighted the role of aberrant glycosylation in B7-H3 stabilization in TNBC, protecting the molecule from ubiquitination [141]. In mouse models of TNBC, the inhibition of B7-H3 via monoclonal antibodies has been shown to convert immune-cold tumors into immune-hot tumors, thereby enhancing the effectiveness of anti-PD-L1 therapy [140]. Similarly, Interventions aimed at inhibiting abnormal B7-H3 glycosylation improved responses to anti-PD-L1 therapy [141]. What is more, silencing B7-H3 expression has been found to enhance the sensitivity of cancer cells to mTOR inhibitors and paclitaxel by disrupting the JAK2/STAT3 signaling pathway [140,142], while B7-H3-induced activation of the Raf/MEK/ERK signaling has been implicated in promoting lung metastasis [143].

B7-H3 was shown to serve as a negative prognostic factor, correlating with poor patient outcomes in breast cancer involving reduced OS, RFS, and PFS [144]. In TNBC, B7-H3 expression in stromal cells has also been associated with adverse DFS and RFS [145]. The observed correlation between high B7-H3 expression and the aggressive phenotype of breast cancer has prompted further preclinical and clinical investigations into therapeutic agents targeting B7-H3. Enoblituzumab, an anti-B7-H3 monoclonal antibody, is currently under investigation in patients with TNBC, either as a monotherapy or in combination with anti-PD-L1 agents [146]. Additionally, CAR T-cell therapy is being tested for application in breast cancer treatment [147].

### 3.5. Cervical Cancer—Molecular Mechanisms of B7-H3 Influence on the Disease

In cervical cancer, B7-H3 expression has been found in both tumor and stromal cells, and it was associated with advanced disease stages, increased tumor sizes, and shorter OS [148]. Additionally, serum levels of B7-H3 in patients were significantly elevated [148]. In cervical squamous cell carcinoma, positive staining for B7-H3 was observed in 73% of patients. In contrast, only 16% of clear cell carcinoma cases exhibited B7-H3 expression, which was not correlated with any clinicopathological features or prognosis [149]. MicroRNA-199a has been shown to directly target B7-H3 mRNA in cancer cells, leading to decreased cell proliferation, migration, and invasiveness by downregulating the B7-H3-activated AKT/mTOR pathway. Levels of microRNA-199a were significantly lower in cervical cancer tissues compared to adjacent normal tissues, highlighting its role in regulating B7-H3 expression [69]. In vitro studies have demonstrated that B7-H3 enhances the self-renewal capacity of cervical cancer stem cells and contributes to chemoresistance against cisplatin [150]. Finally, B7-H3 has been implicated in fostering an immunosuppressive tumor microenvironment by increasing the production of IL-10 and TGF-β through the stimulation of the p-JAK2/STAT3 signaling pathway in vivo [151].

### 3.6. Ovarian Cancer—Prognostic Role of B7-H3 and Its Influence on Immune Responses

The upregulation of B7-H3 expression in ovarian cancer has been associated with a decreased number of IFNγ+ CD8+ T cells, enhanced tumor aggressiveness, and higher risk of metastasis. Kovaleva et al. reported that patients exhibiting serum levels of sB7-H3 at or above 11.6 ng/mL demonstrated significantly worse overall survival. Additionally, the overexpression of B7-H3 correlates with patient age, with markedly elevated levels observed in individuals aged over 60 years. B7-H3 expression was found to be positively regulated by Golgi membrane protein 1 (GOLM1), which enhances the secretion of soluble B7-H3. 

Within the ovarian cancer tumor microenvironment, B7-H3 facilitates tumor growth by promoting macrophage polarization towards the M2 phenotype. Subsequently, it induces angiogenesis, tissue remodeling, and the suppression of immune responses, particularly through the enhancement of the CCL2–CCR2–M2 macrophage axis. The inhibition of B7-H3 expression has been shown to result in a reduced population of M2 macrophages alongside an increased presence of CD8+ T cells. Moreover, B7-H3 contributes to the establishment of an immunosuppressive tumor microenvironment by attenuating the functionality of NK cells. In contrast, the inhibition of B7-H3 via miR-29c restores the anti-tumor activity of NK cells and improves survival outcomes in ovarian cancer murine models [55].

### 3.7. Prostate Cancer—Prognostic and Predictive Potential of B7-H3

B7-H3 has been found to be abnormally expressed in prostate adenocarcinoma as well as in benign prostatic epithelial tissues. However, the levels of B7-H3 were significantly lower in benign tissues compared to cancerous ones [152]. The presence of B7-H3 has been confirmed in both castration-resistant prostate cancer (CRPC) and hormone-sensitive prostate cancer (HSPC). A study conducted by Guo C et al. found that 93% of biopsies from CRPCs and 97% of corresponding HSPC samples were positive for B7-H3 staining [153].

The expression of B7-H3 positively correlated with aggressive histological features, the presence of metastasis, and an increased risk of progression after surgical treatment [152]. B7-H3 was upregulated in prostate cancer tumors with defects in DNA repair genes, including ATM and BRCA2, and was associated with high expression levels of ERG and androgen receptors (ARs) [153]. During intensive neoadjuvant hormone therapy, B7-H3 concentrations significantly decreased; however, in metastatic castrate-resistant prostate cancer (CRPC) that is resistant to Enzalutamide, B7-H3 expression remained elevated and contributed to resistance mechanisms [37]. Research has shown that the B7-H3 promoter exhibits increased transcriptional activity and contains a potential AR binding site, suggesting that AR may play a role in regulating B7-H3 expression in CRPC [37]. A study by Kang N et al. demonstrated that B7-H3 expression in PCa patient-derived xenograft (PDX) models is negatively regulated by AR during the early phase of androgen deprivation therapy (ADT) treatment but is positively associated with PCa proliferation during later stages of disease progression. These findings suggest that B7-H3 could serve as a biomarker for diagnosis, prognosis, and responses to ADT. Furthermore, combining ADT with B7-H3-targeting immunotherapy might be beneficial for treating hormone-naïve PCa to prevent fatal relapses into CRPC [154]. B7-H3 was found to be the most overexpressed immune checkpoint in prostate tumors with PTEN and TP53 inactivation, which contributes to a worse outcome [155]. The proposed underlying mechanism is that the loss of PTEN and the inactivation of TP53 activate the transcription factor Sp1, leading to increased B7-H3 expression [155]. Similar to other malignancies, microRNAs (miRNAs) also play a role in regulating B7-H3 expression in prostate cancer. miR-187 has been shown to downregulate B7-H3 and reduce the JAK3-STAT3-Slug signaling pathway involved in cancer progression. Restoring miR-187 expression effectively targeted B7-H3, reducing its expression levels and repressing the aggressive phenotype of PCa cells [156].

B7-H3 may also gain increasing attention as a fluorescent biomarker for accurately localizing prostate cancer tissue during prostatectomy. Tian Y. et al. developed a fluorescent probe (Ab_B7−H3-800CW) using a B7-H3 monoclonal antibody, which effectively images PCa cells in vivo. This method may enhance the detection accuracy of prostate cancer lesions and metastatic lymph nodes during surgery, leading to more precise resections and better surgical outcomes [157].

Currently, multiple therapeutic modalities targeting B7-H3 in prostate cancer are under investigation. The antibody–drug conjugate DS-7300a, which targets B7-H3, has shown a significant decrease in tumor size in mouse models of castrate-resistant prostate cancer (CRPC) and neuroendocrine prostate cancer (NEPC) [154]. B7-H3 CAR T-cells have exhibited significant cytotoxicity against PC3 and LNCaP prostate cancer cell lines in mouse models.

### 3.8. Renal Cancer—Molecular Mechanisms, Possible Prognostic and Predictive Role, and Therapeutic Opportunities Related to B7-H3

Numerous studies have indicated that the immunomodulatory molecule B7-H3 is significantly upregulated in renal cell carcinoma (RCC), exhibiting the highest expression levels in urothelial and sarcomatoid carcinoma subtypes [69,158,159]. In silico analyses have demonstrated that among the B7 family proteins featuring aberrant expression in renal malignancies, B7-H3, B7H5, and B7H7 are most prominently expressed in clear cell renal cell carcinoma (ccRCC) when compared to normal renal tissue [57]. Saeednejad Zanjani et al. reported that in a cohort of 225 RCC patients, 218 (96.9%) exhibited positive membranous B7-H3 staining, while 178 (79.1%) showed cytoplasmic expression. B7-H3 was localized in tumor cells, the surrounding stroma, and tumor vascular endothelium [160]. Notably, Inamura et al. found that only 37 out of 252 ccRCC cases (15%) demonstrated high CD276 expression within tumor cells; however, 137 samples (54%) displayed elevated expression levels in the tumor vasculature. Furthermore, B7-H3 upregulation was observed in CD14+ monocytes within tumors relative to normal kidney tissue [15]. In ccRCC, the expression of CD276 displayed a correlation with CTLA-4 and PD-L1, and in the vascular endothelium, it coexpressed with Tie-2, a receptor involved in angiogenic processes [158]. Saeednejad Zanjani et al. did not identify a correlation between membranous B7-H3 expression and clinicopathological parameters; however, cytoplasmic B7-H3 expression was associated with several aggressive features, including lymph node and Gerota's fascia invasion, histological tumor necrosis, tumor size, and tumor grade—showing the highest expression levels in grade IV tumors [160]. Interestingly, Deng et al. reported a decrease in B7-H3 expression in older patients [161], while elevated CD276 levels within tumor vasculature were linked with older age according to Inamura et al. [15]. The upregulation of B7-H3 in the tumor vascular endothelium correlated with more advanced ccRCC grade and tumor-node-metastasis (TNM) stage and was positively associated with microvessel density (MVD), supporting the notion that CD276 may play a crucial role in angiogenesis [162]. Similarly, B7-H3 expression in CD14+ monocytes was correlated with advanced TNM stages and tumor grades. It has been reported that men exhibit higher B7-H3 expression in ccRCC than women; however, this finding has not been consistently validated across studies [57]. Furthermore, patients with lower B7-H3 expression had better objective response rates and longer durations of response to nivolumab, indicating the potential role of CD276 in therapy resistance [163]. Several investigations have linked elevated B7-H3 expression with poorer prognostic outcomes in RCC, associating it with diminished overall survival (OS), disease-specific survival (DSS), and shorter disease-free survival periods [69,164]. Increased expression of B7-H3 in tumor vasculature has been independently linked to higher overall mortality rates. Inamura and colleagues noted that elevated CD276 expression, whether in tumor cells or vasculature, was associated with adverse prognostic outcomes primarily in the context of tumors exhibiting high FOXP3+ cell densities, suggesting that B7-H3 may exert its protumor and immunosuppressive effects in RCC predominantly via FOXP3+ regulatory T cells [15]. Notably, in RCC patients treated with nivolumab, high B7-H3 expression was identified as the sole factor associated with survival, correlating with shorter OS and progression-free survival (PFS) [163]. Hence, B7-H3 has the potential to serve as a biomarker for response to immunotherapy in RCC patients, warranting consideration of B7-H3-targeting therapies in conjunction with existing treatment modalities [163]. There remain relatively few studies addressing the influence of CD276 on immune responses in RCC. Reports have shown a positive association between B7-H3 expression in tumor cells and vasculature with FOXP3+ cell infiltration. Given the critical role of these cells in mediating B7-H3-induced inhibition of anti-tumor activity in ccRCC, dual targeting of B7-H3 and FOXP3+ cells may represent a promising therapeutic avenue [15]. Additionally, B7-H3-high tumors have been observed to present increased scores for T-cell exhaustion, macrophage activity, and myeloid immune evasion. Further, the augmented expression of this molecule was connected to disruption in TNF, IL-2, and NK-κB signaling pathways. Therefore, B7-H3 might facilitate a pro-tumorigenic environment in RCC by impairing immune responses, promoting T-cell exhaustion, and recruiting tumor-associated macrophages (TAMs); however, such findings necessitate further elucidation [15]. Overall, more comprehensive research is imperative to clarify the influence of elevated B7-H3 expression on the immunological landscape in RCC.

Experimental silencing of B7-H3 has unveiled various anti-cancer effects in RCC models. Zhang et al. demonstrated that B7-H3 in cancer-associated fibroblasts (CAFs) plays a role in inhibiting CAF apoptosis while stimulating secretion of hepatocyte growth factor (HGF) and stromal cell-derived factor-1 (CXCL12). Silencing B7-H3 led to increased apoptosis in CAFs, accompanied by decreased proliferation, migration, and invasion. Moreover, the injection of B7-H3-silenced CAFs in murine models resulted in reduced tumor volumes relative to controls [69]. Additionally, Xie and colleagues illustrated that B7-H3, through its interactions with fibronectin, can activate the PI3K/AKT and p38/ERK signaling pathways, thereby promoting epithelial–mesenchymal transition (EMT) [69]. Conversely, knockdown of B7-H3 was shown to decrease the migratory and invasive capacities of ccRCC cells [57]. Furthermore, B7-H3 silencing enhanced sensitivity to the tyrosine kinase inhibitor axitinib in RCC cell lines, indicating that targeting B7-H3 could yield synergistic effects when combined with established therapies [57].

Several strategies targeting B7-H3 have been proposed for the treatment of renal cell carcinoma (RCC). Sun et al. investigated the effects of the H1-pHMGB1 (high mobility group box 1 protein)/pB7-H3 vaccine in a renal carcinoma model. Their study revealed that immunization led to significant tumor growth inhibition, an increase in CD11c+ dendritic cells (DCs), and the induction of CD8 T-cell antitumor cytotoxicity [165]. Similarly, Zheng et al. reported promising results using an adenovirus vaccine aimed at B7H1 and B7-H3. Additionally, some researchers have proposed the use of B7-H3 CAR T cells, which demonstrated high efficacy and specificity in eliminating RCC cells, inhibiting tumor growth, and prolonging the survival of tumor-bearing mice [165]. These findings suggest that targeting B7-H3 could represent a promising strategy against RCC, highlighting the need for further studies in this area.

### 3.9. Bladder Cancer and Urothelial Carcinoma (UCC)—Disease Aggressiveness and Therapeutic Options Related to B7-H3

A recent meta-analysis found that B7-H3 levels were significantly higher in bladder cancer tissues compared to non-tumor tissues [166]. However, research conducted by Koyama and colleagues demonstrated that only 36 out of 271 cases (13%) of upper tract urothelial carcinoma (UTUC) exhibited positive IHC staining for B7-H3 [167]. Azuma et al. further reported that serum levels of soluble B7-H3 (sB7-H3) were increased in 47% of patients with non-muscle invasive bladder cancer (NMIBC), compared to just 8% in healthy individuals [167]. In bladder cancer, a systematic review and meta-analysis by Sun et al. revealed a significant association between B7-H3 expression and factors such as age, depth of infiltration (Ta-2 vs. T3-4), and more advanced tumor stages in muscle-invasive bladder cancer [166]. On the contrary, no significant correlation was observed between B7-H3 expression and lymph node metastasis or tumor stage in NMIBC. Furthermore, in another study, high levels of B7-H3 were linked to increased vascular invasion and distant metastasis in urothelial cancer of the bladder (UCB) [58]. In UTUC, B7-H3 expression was associated with higher tumor grades, greater T stages, and the presence of lymph node metastasis [167]. Additionally, Deol and colleagues found that the complete pathologic response (pCR) rate to neoadjuvant platinum-based chemotherapy was higher in the B7-H3-low group compared to the B7-H3-high group among MIBC patients, suggesting that B7-H3 may play a role in chemotherapy resistance [168]. These findings indicate that elevated B7-H3 expression in UCC and bladder cancer is likely associated with a more aggressive disease course, although the available data is still limited. In the meta-analysis by Sun et al., no significant association was found between B7-H3 expression and patient survival in bladder cancer. However, the authors noted limitations in the existing data [166]. In UTUC, positivity for B7-H3 correlated with shorter cancer-specific survival (CSS) and metastasis-free survival (MFS), with even poorer outcomes noted in cases of co-positivity for B7-H3 and PD-L1 [167]. Moreover, elevated levels of sB7-H3 were associated with worse relapse-free survival (RFS) and progression-free survival (PFS) in NMIBC, indicating its potential as a prognostic marker [169]. The role of B7-H3 in UCC and bladder cancer is still not fully understood. There is limited information regarding its influence on the immune landscape of tumors. Xu et al. demonstrated a positive correlation between B7-H3 expression and the infiltration of CD163+ tumor-associated macrophages (TAM) in UCB, suggesting that B7-H3 may have immunosuppressive effects [154]. Additionally, B7-H3 may enhance bladder cancer cell invasiveness through the upregulation of MMP2 and MMP9 as well as the activation of PI3K/Akt/STAT3 signaling pathways. Consistently, its knockdown inhibited cancer cell invasion and migration and reduced tumor volume in mouse models [170]. Therefore, B7-H3 has the potential to be an important target for bladder cancer treatment. Further research into its immune and non-immune functions is encouraged to clarify its role in the disease.

### 3.10. Head and Neck Cancers—Conflicting Results and Potential Therapeutic Strategies Involving B7-H3

In head and neck cancers, B7-H3 has been shown to exhibit pleiotropic activity by reshaping the immune microenvironment and influencing non-immunological processes, such as the maintenance of cancer stem cell populations and the inducing epithelial-to-mesenchymal transition. Studies evaluating B7-H3 expression in HNSCC have yielded conflicting results. Boschert et al. reported that expression levels of B7-H3 across six HNSCC cell lines were comparable to those of PD-L1, although certain cell lines exhibited negative B7-H3 expression [171]. Conversely, other investigations have documented significantly elevated and stable overexpression of B7-H3 in HNSCC cells [172]. In a study analyzing B7-H3 expression in a cohort of 408 HNSCC patients, tumors exhibited pronounced staining for B7-H3, and its overexpression was correlated with improved survival outcomes in HPV-negative HNSCC suggesting antitumoral activity of B7-H3 in this subgroup of patients [173]. Notably, B7-H3 has been found to co-express with various immune checkpoints, including PD-L1, IDO-1, PD-1, TIM-3, LAG-3, and VISTA. This co-expression leads to distinct patterns characterized by specific signatures of immune molecules [174]. In oral tongue squamous cell carcinoma (OTSCC), B7-H3 overexpression was associated with decreased survival rates exclusively in the subgroup of immune "hot" tumors characterized by increased T-cell infiltration [175]. B7-H3 contributes to an immunosuppressive landscape in HNSCC by directly promoting tumor cell immune evasion and enhancing the infiltration of immunosuppressive cells such as myeloid-derived suppressor cells (MDSCs) and tumor-associated macrophages (TAMs) [39]. Moreover, modifications in B7-H3 glycosylation have been found to remodel the immune microenvironment, subsequently facilitating tumor progression in oral squamous cell carcinoma (OSCC) via enhanced binding efficacy of B7-H3 to DC-SIGN (DC-specific intercellular adhesion molecule 3) and Langerin on immune cells [176]. In hypopharyngeal squamous cell carcinoma, high B7-H3 expression demonstrated a negative correlation with T-cell infiltration and was positively associated with a heightened risk of metastasis and reduced disease-specific survival during follow-up [177]. Research by Wang et al. indicated that cancer stem cells (CSCs) in squamous cell carcinoma preferentially overexpress B7-H3 on their surface, usurping PD-L1 as a mechanism to evade host immune surveillance. The silencing of B7-H3 expression through targeted antibodies resulted in a reduction in the CSC population, facilitated the activation of CD8+ T cells, and subsequently diminished tumor growth and lymph node metastasis in a mouse model of HNSCC. Furthermore, inhibiting B7-H3 influenced the CSC phenotype by mitigating the epithelial–mesenchymal transition [21]. In OSCC, B7-H3 has been shown to support tumor growth by inducing aerobic glycolysis in cancer cells through upregulating the PI3K/Akt/mTOR signaling pathway [176]. Similar to other malignancies, the expression of B7-H3 in HNSCC is tightly regulated by microRNAs, which are themselves modulated and degraded by long noncoding RNAs (lncRNAs) that competitively bind to targeted microRNAs. The upregulation of LINC01123, which targets microRNA regulating B7-H3, miR-214-3p, leads to the inhibition of CD8+ T-cell activation, thereby promoting tumor growth through the upregulation of B7-H3 expression in HNSCC [178]. Finally, an increasing number of therapeutic modalities are targeting B7-H3 in head and neck cancers. Dong et al. demonstrated that a bispecific antibody–drug conjugate (BsADC) targeting both B7-H3 and PD-L1, and delivering auristatin E, a chemotherapeutic agent, exhibited antitumor effects in laryngeal squamous cell carcinoma cell lines and mouse models. In vitro experiments indicated that this BsADC promoted tumor-specific immunity and activated immunogenic cell death (ICD), a process involving alterations in the antigens expressed by cancer cells that elicit immune responses and enhance recognition of dying cells by T lymphocytes [179].

## 4. B7-H3 as a Target

### 4.1. CAR-T Therapy

A Chimeric Antigen Receptor (CAR) is a protein composed of an extracellular domain, a transmembrane, and an intracellular signaling-activation domain. While extracellular domain binds to antigens, transmembrane domain secures the CAR to the effector cell membrane. Chimeric antigen receptor T cell (CAR-T) involves redirected T cells against tumor antigens through the engineered expression of Chimeric Antigen Receptors (CARs) to eliminate tumors. T lymphocytes, either autologous or allogeneic, undergo genetic modification with CARs designed to target a particular tumor antigen pivotal in tumor advancement. With this modification, modified T cells proficiently identify and eradicate specific cancer cells without relying on the major histocompatibility complex (MHC) antigen [180]. In various preclinical studies across multiple cancer types, CAR-T therapy against B7-H3 has demonstrated significant promise [181,182]. What is more, B7-H3-CAR-T therapy has been reported to exhibit strong anti-tumor effectiveness in many tumor types [84,102,182,183,184]. There are many ongoing clinical trials validating the effectiveness of CAR-T therapy directed at B7-H3 due to its clinical potential [185,186,187,188,189,190,191]. On the other hand, its low-level expression in some non-tumor tissues induces the risk of off-target [192,193]. Preclinical studies have demonstrated that CAR T-cells, especially those employing highly sensitive nanobody-based constructs, are capable of inducing systemic negative effects even in the absence of tumor, with symptoms including weight loss, hematologic abnormalities, and T-cell infiltration into healthy organs such as bone marrow, liver, and spleen [192]. Adverse events of CAR T-cell therapy include not only those related with off-tumor toxicity, but also, one of the most common, Cytokine Release Syndrome (CRS) with symptoms that can range from mild to life-threatening [100]. Toxicities of CAR T-cell therapy include Cytokine Release Syndrome (CRS), one of the most common adverse events (AEs) with symptoms that can range from mild to life-threatening [194]. The wide spectrum of neurotoxicity, following CAR T-cell therapy ranging from encephalopathy to seizures, obtundation, and possible death, was also observed. Moreover, CAR T-cell therapy frequently led to central nervous system immune activation, characterized by transient headache, fever, and cerebrospinal fluid pleocytosis—indicative of local inflammatory responses. However, dose-limiting adverse events were not observed [100]. The pathophysiology of immune-effector-cell-associated neurotoxicity syndrome (ICANS) is poorly understood, and neurological events may occur independently of CRS-related toxicities [195]. A risk–benefit balance must be carefully evaluated in clinical trials.

### 4.2. ADC Therapy

Antibody–drug conjugates (ADCs) are an innovative therapeutic strategy for resistant cancers [196]. ADCs merge the target specificity of a monoclonal antibody (mAb) with cytotoxic agents or radionucleotides, facilitating the targeted delivery of these agents to tumors and enhancing therapeutic efficacy. The preclinically developed ADC, MGC018, featuring a duocarmycin payload and targeting B7-H3, has demonstrated encouraging anti-tumor activity in preclinical models across diverse cancer types [197]. In addition, DS-7300a, a different ADC with targeting capabilities, incorporates a B7-H3-targeting monoclonal antibody with a DNA topoisomerase I inhibitor. This ADC demonstrates strong anti-tumor effects against B7-H3 positive tumors [198]. There have been several clinical trials with ADCs such as Vobramitamab duocarmazine (MGC018) [199] and DS-5573a [200]. Moreover, there are still some ongoing studies on Ifinatamab Deruxtecan (I-DXd) [201], HS-20093 [202], DB-1311 [203], and Vobramitamab duocarmazine (MGC018) [204].

Regardless of their therapeutical potential and improved safety profiles, ADCs are still reported to cause grade 3 and/or grade 4 toxicity [205]. Hematologic, hepatic, neurologic, ophthalmic, and pulmonary-treatment-related effects have been observed irrespective of targeted antigen [206]. The mentioned toxicities are mediated by any of the components of the drug. Despite the fact that the majority of AEs result from premature release of the payload into bloodstream or tumor microenvironment, noncancerous cells expressing B7-H3 antigen are also capable of binding ADC [206]. Therefore, ADC’s safety profile may be influenced by the B7-H3 expression pattern, with adverse effects potentially occurring through both on-target and off-target mechanisms [207].

B7-H3-directed ADCs, vobramitamab duocarmazine, and ifinatamab deruxtecan caused dose-limiting neutropenia and fatigue. Other ADCs were associated with AEs like neutropenia, anemia, pyrexia, nausea, thrombocytopenia, hypoalbuminemia, vomiting, lymphopenia, infusion-related reaction, fatigue, and palmar–plantar erythrodysesthesia [208].

### 4.3. ADCC Therapy

Antibody-dependent cellular cytotoxicity (ADCC) is a crucial mechanism of action of therapeutic monoclonal antibodies (mAbs). This mechanism, called antibody-dependent cellular cytotoxicity, involves recognizing and eliminating target cells by immune effector cells through Fc receptors. This process occurs when the antibody binds to an antigen on the cancer cell's surface using a Fab fragment while simultaneously binding to the Fc receptor on the effector cell, which initiates a cytotoxic reaction [209]. The Fc region binds to Fc receptors (FcR) on the surface of cytotoxic cells [210].

Enoblituzumab (MGA271) is the most studied IgG1κ B7-H3 monoclonal antibody mediating antibody-dependent cellular cytotoxicity. Enoblituzumab specifically targets B7 Homolog 3 (B7-H3). It has been engineered to improve binding by activating the Fc-gamma receptor on immune cells while minimizing its interaction with the inhibitory Fc-gamma receptor. The NCT02475213 trial assessed enoblitizumab and pembrolizumab or retifanlimab in patients with advanced solid tumors, including patients with various tumors expressing B7-H3 such as NSCLC, melanoma, squamous cell cancer of the head and neck, and urothelial cancers [211]. According to available results, this drug was well tolerated. However, as for the other therapies, their toxicity profile must be carefully evaluated due to the low-level expression of B7-H3 on some normal tissues [212]. Although the treatment-related adverse events (TRAEs) were reported by most patients (87.2% in a study conducted by Aggarwal et al.), grade ≥3 toxicities occurred in a limited number of patients (28.6%) [212]. The most common side effects observed during clinical trials were fatigue, neurological symptoms such as dizziness, paresthesia, flu-like symptoms, and gastrointestinal symptoms such as nausea, vomiting, anorexia, and constipation [212,213].

### 4.4. Monoclonal Antibody (mAb) Therapy

Monoclonal antibody therapeutics (mAbs) have been approved for various targets and diseases and play a crucial role in cancer treatment by targeting antigens expressed by tumors [214]. Identifying suitable tumor antigens is essential for the effective development and application of mAb-based immunotherapies as these antigens are vital targets for these treatments [215]. They can operate through multiple mechanisms such as direct binding to antigens, interference with antigen-antibody interactions, and facilitating antibody-dependent cellular cytotoxicity (ADCC) [215]. The effectiveness of mAb therapy, which targets PD-1, PD-L1, and CTLA-4, was implemented to use B7-H3 mAb therapy for tumors expressing elevated levels of B7-H3 [86,216]. What is more, blocking B7-H3 with mAbs has not only demonstrated enhanced infiltration of CD8+ T cells and NK cells into tumors but has also led to diminished tumor growth and, most importantly, extended survival in murine models, offering a ray of hope for cancer treatment outcomes [29,216,217].

Omburtamab (8H9) is a murine anti-B7-H3 IgG1 monoclonal antibody (mAb) that has been extensively studied in various clinical trials [218,219,220]. One particular trial NCT03275402 investigated compartmental radioimmunotherapy (cRIT) using omburtamab, administered intraventricularly, to treat metastatic and recurrent tumors of the central nervous system (CNS). The study was terminated during phase I, and the most common adverse events reported included lymphopenia, intracranial hemorrhage, and decreased platelet count [220].

Despite the fact that anti B7-H3 mAbs present promising antitumor activity, their clinical development requires thorough evaluation of safety profile and potential adverse events. These concerns have been addressed by multiple preclinical and clinical studies with favorable outcomes.

Reported toxicities were mostly limited to mild-to-moderate infusion-related reactions and transient constitutional symptoms such as headache and fever. What is more, occasional thrombocytopenia as one of the hematologic effects was observed [108,208]. Overall, these therapies were well tolerated, with rare or absent dose-limiting toxicities, even with higher doses [208]. Additionally, engineered Fc-enhanced antibodies (such as enoblituzumab) have shown minimal reactivity to non-tumor tissues, which reduces the risk of off-tumor toxicity. The absence of major organ damage during anti B7-H3 mAbs therapy has been also documented [210]. Moreover, inflammatory models have shown that mAbs targeting this B7 family member display immunomodulatory activity, including suppression of proinflammatory cytokines and NF-κB signaling. While potentially beneficial, these effects require careful immune monitoring [221].

### 4.5. Radioimmunotherapy

Radioimmunotherapy (RIT) is another modern treatment method that allows for the targeted localization of radionuclides in specific cancer cells while sparing healthy tissues from the harmful effects of radiation [220].

After administration, the radiolabeled conjugate is retained in tissues expressing the specific antigen or receptor while being cleared from those that do not. This method utilizes a particle-emitting radionuclide linked to a carrier molecule, usually an antibody or an antibody fragment. This molecule binds to an overexpressed antigen or receptor in cancer cells [222]. The most commonly used carrier in radioimmunoconjugates is omburtamab (8H9). It is distinct from other antibodies that target B7-H3 because it binds to the FG loop of B7-H3, a critical area for its immunological function.

One trial, NCT01099644, investigated compartmental radioimmunotherapy using omburtamab in patients over one year old with desmoplastic small round cell tumors and other solid tumors affecting the peritoneum. The study was terminated during phase I, and the major related adverse events were transient neutropenia, thrombocytopenia, and pain lasting < 2 h related to saline infusion [223].

On the other hand, other preclinical and early clinical studies suggested that B7-H3 targeted RIT is promisingly well tolerated and associated with a manageable toxicity profile [208,222,224]. Studies using radiolabeled mAbs, mostly 8H9, but also with 212Pb-labeled antibodies, have demonstrated limited off-target accumulation with highly selective tumor uptake, especially through intraventricular and intrathecal administration.

Additionally, favorable dosimetry and reduced systemic exposure, translating into minimal organ or hematological toxicity, have been documented. For instance, no dose-limiting toxicities were observed and reported adverse events restricted to transient symptoms. Patients gave commonly history of nausea, fever, and headache during therapy [208].

Nonetheless, the use of murine antibodies still exhibits a risk of immunogenicity, which may limit repeated dosing. Development of fully human or humanized antibodies may result in a reduction in this risk occurring in future clinical applications [224].

**Figure 1:**
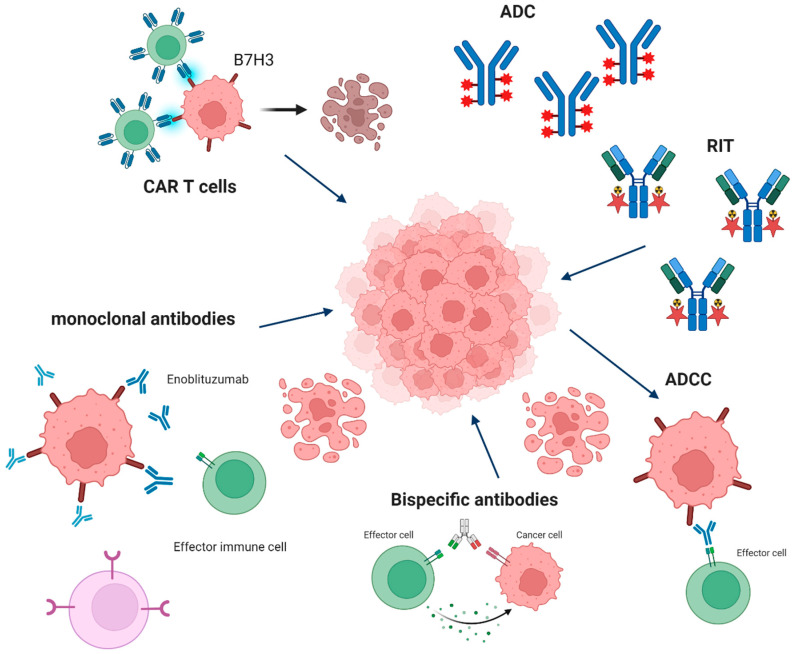
Therapeutic modalities aimed at B7-H3 inhibition.

Different strategies for B7-H3 targeting in cancer treatment are illustrated in Figure 1, Table 2, Table 3 and Table 4 present ongoing clinical trials, while Table 5 presents completed trials involving B7-H3 blockade, respectively. The expression rates of B7 family proteins in the discussed tumors are shown in Table 4.

### 4.6. Potential Mechanisms of Resistance to B7-H3-Targeted Therapies

Although B7-H3 has emerged as a promising target for immunotherapeutic strategies across numerous solid tumors due to its limited expression in normal tissues and high prevalence in malignancies, resistance to B7-H3 targeted therapies is more commonly recognized obstacle, limiting long-term efficacy [225]. Current evidence suggests that this resistance arises through diverse and overlapping biological processes, including epigenetic regulation, tumor-intrinsic alternations, immunosuppressive microenvironments, and pharmacologic or design-related limitations of the therapeutic agents [225].

Tumor cells have been observed to downregulate B7-H3 or present nonuniform antigen expression, which enables those cells to evade immune pressure, especially in the context of CAR T-cell therapy. This phenomenon has been observed to contribute to disease recurrence [226]. What is more, CAR T-cells targeting B7-H3 therapies face several specific limitations, such as antigen escape, T-cell exhaustion, potential off-tumor toxicity, and tonic signaling [192].

Additionally, expression of B7-H3 is also downregulated through aberrant DNA methylation, particularly in RB1-deficient and neuroendocrine prostate cancers. This component of epigenetic silencing impairs efficacy of B7-H3 targeted ADCs [227]. Nonetheless, tumors B7-H3-positive exhibit resistance to therapies using ADCs due to intrinsic deficiencies in processing and responding to cytotoxic payloads. Susceptibility to DNA-damaging agents is contingent on SLFN11 expression, TP53 status, and replication stress. The absence of these factors has been associated with therapeutic resistance [225].

The crucial role in mediating resistance to therapies has been also assigned to the tumor microenvironment. Elevated ITGB6 expression promotes the recruitment of PF4^+^ macrophages via the CXCL1-CXCR1 axis, limiting effective CD8^+^ T-cell infiltration and response to anti B7-H3 antibodies [228]. Moreover, CAR T-cell migration may be impaired by IL-8, secreted by tumor cells, although engineering CAR T-cells to express chemokine receptors, including CXCR2, has proved to promisingly overcome this barrier [226].

Immune evasion is also promoted via histone lactylation and through the STAT3-ULBP2 axis [229]. Tumor-associated metabolic changes, such as lactate accumulation, drive histone lactylation, resulting in enhanced B7-H3 transcription in TME. Accordingly, T-cell activation suppression has been observed. This mechanism contributes to both immune-cold phenotype and limited efficacy to B7-H3 targeted agents, including monoclonal antibodies, CAR T-cells, and immune checkpoints inhibitors [229]. Furthermore, activation of STAT3 signaling arising from B7-H3 overexpression leads to downregulation of ULBP2, a crucial ligand for NKG2D^+^ cytotoxic lymphocytes, resulting in suppression of Vγ9Vδ2 T cell–mediated cytotoxicity. Hence, tumors are capable of evading immune-based depending on γδ T-cell activity or NK cell engagement therapies [230]. Modulation of histone lactylation and pharmacological inhibition of STAT3 can restore immune sensitivity in B7-H3-mediated resistance, promisingly highlighting potential of combining targeted or epigenetic therapies with immunotherapeutic approaches to overcome tumor immune evasion [229,230].

Enhanced B7-H3 expression in tumors may promote abnormal survival under oxidative or chemotherapeutic stress by forming a complex with inosine monophosphate dehydrogenase 2 (IMPDH2), an enzyme that plays a critical role in purine nucleotide biosynthesis. This interaction facilitates enhanced purine production, stabilizes nucleotide production, and promotes DNA repair independently of immune involvement, contributing to therapy resistance. Such metabolic adaptation diminishes the effectiveness of conventional cytotoxic agents, including DNA-damaging drugs [231].

### 4.7. Combined Therapies

While monotherapy approaches targeting B7-H3 often face restrictions due to tumor heterogeneity, immune evasion, and a suppressive tumor immune microenvironment, recent efforts have increasingly focused on combining B7-H3 directed modalities with other molecular or immune interventions to improve tumor selectivity, durability, and efficacy.

Latest studies focused on B7-H3×CD3 bispecific T-cell engagers (BiTEs) gave evidence of a promising modality capable of redirecting T cells to tumor cells. However, limited half-life and systemic toxicity have driven innovations including lipid nanoparticle (LNP)-delivered mRNA encoding B7-H3 × CD3 BsAbs, enabling robust in vivo expression and sustained antitumor responses [79]. Combined ICAM-1-targeted ADCs with B7-H3 × CD3 BsAbs is another innovated approach, which both enhances tumor cell killing and remodels the TIME through activation of dendritic cells, promoting CD8+ T-cell infiltration and reducing immunosuppressive cell populations such as MDSCs and Tregs [232].

In addition to CD3-based BsAbs, co-stimulatory bispecific constructs, such as B7-H3 × CD28 and B7-H3 × 4-1BB, have been invented to amplify T-cell responses. Thus, a B7-H3 × CD28 bispecific antibody, XmAb808, synergizes with anti-PD-1 and CD3-engaging therapies, minimizing systemic activation while increasing T-cell activation and tumor infiltration [233]. Furthermore, B7-H3 × 4-1BB BsAbs present selective activation of terminally differentiated CD8+ TILs within TME, overcoming dose-limiting toxicities, associated over past years with systemic 4-1BB agonists. Remarkably, co-administration with PD-1 blockade leads to enhanced antitumor efficacy [234].

Nonetheless, engineered to improve tumor selectivity, bispecific targeting dual agents, such as GD2 × B7-H3, address the challenge of off-tumor toxicities typical in anti-GD2 therapies through restriction of T-cell engagement to cells expressing both markers, hence reducing peripheral nerve binding and enhancing therapeutic index [105].

The clinical success of ADCs has driven the development of B7-H3-directed ADCs, including dual-payload constructs, combining cytotoxic agents with immune activation in preclinical models of triple negative breast cancer, indicating a combined therapeutic strategy targeting both tumor cells and the immunosuppressive microenvironment [235]. The combination of ADCs with immunotherapies, including checkpoint inhibitors or bispecific antibodies, supports a dual-targeting strategy addressing both tumor mass and immunosuppressive mechanisms.

In neoplasms commonly resistance to radiation and immune escape, for instance, in chondroma, the combination of B7-H3 CAR T-cells with irradiation not only improved cytotoxicity against tumor mass but also depleted radiation-resistant cancer stem-like cells, which is indicative of synergistic mechanism [236]. Further enhancement was demonstrated in rhabdomyosarcoma by combining B7-H3 targeting with dual-CAR constructs also directed at FGFR4, providing improved clearance over single-target approaches.

Despite B7-H3 classification as an immune checkpoint, its function appears more common with immune evasion and resistance. Hence, clinical promise of the combination of B7-H3 inhibition with other immune checkpoint inhibitors, including PD-1 and CTLA-4, has been observed. Recent studies have reported encouraging efficacy of co-administration of enoblituzumab (anti B7-H3) and pembrolizumab (anti PD-1), highlighting potential for synergistic immune activation [212]. Additionally, dual inhibition of B7-H3 and CTLA-4 or PD-L1 promoted curative responses in murine models of castration-resistant prostate cancer, emphasizing the value of checkpoint combination strategies within molecularly defined tumor subtypes [155].

Short serum half-life and systemic toxicity have driven the development of innovative delivery platforms such as mRNA and nanoparticle-based systems. One notable advancement involves MMP-2-activable nanoparticles that release B7-H3 bispecific antibodies selectively within the TME, enhancing therapeutic precision, limiting off-target toxicities, and inducing ferroptosis. The combined use of ionizable LNPs to deliver mRNA encoding BsAbs, including B7-H3 × CD3, enables in situ antibody production, sustained systemic levels, and improved biodistribution to both tumor sites and secondary lymphoid organs [79].

Future combination strategies will likely depend on rational, biomarker-guided approaches that target both tumor-intrinsic mechanisms and the immunosuppressive microenvironment. Promising directions include combining multiple immune checkpoints, such as B7-H3 with CD47 or PD-L1-engineering Fc regions, to enhance effector functions and co-targeting signaling pathways like EGFR to overcome resistance [237]. Other innovative strategies focusing on reprogramming the tumor immune landscape with immunomodulatory ADCs or bispecific nanoplatforms, as well as delivering therapies via mRNA, lipid nanoparticles, or protease-activated carriers, are also gaining interest [232]. Together, these innovations, guided by genomic profiling and tumor subtype selection, are expected to play a pivotal role in advancing B7-H3-centered combinatorial immunotherapeutic strategies.

## 5. Potential Biomarkers for B7-H3-Targeted Therapies

Biomarkers predicting B7-H3 expression and responsiveness to B7-H3-targeted therapies have not yet been clearly defined. In our previous study on colorectal cancer, we observed that—unlike PD-L1—B7-H3 expression was not associated with microsatellite instability status (MSI/MSS) [238]. A similar observation was reported by Miller et al. [25], who analyzed over 150,000 cases of various solid tumors and likewise found no significant association between high B7-H3 expression and microsatellite instability.

The relationship between B7-H3 and PD-L1—the most extensively studied member of the B7 family—remains inconsistent across the literature. In the large-scale analysis by Miller et al., encompassing more than 156,000 solid tumors, no significant correlation was observed between the expression of these two immune checkpoints [25]. This suggests that the immune escape pathway mediated by B7-H3 may function largely independently of PD-L1-associated mechanisms. Nevertheless, this does not exclude potential therapeutic benefit from combination strategies involving a dual blockade of B7-H3 and PD-L1, particularly in patients with MSS-type CRC, where conventional immunotherapies based on anti–PD-1/PD-L1 monotherapy demonstrate limited efficacy.

In our own cohort of CRC patients, higher B7-H3 levels correlated negatively with the number of tumor-infiltrating lymphocytes (TILs) [238]. A similar inverse association between B7-H3 expression and TIL density has been documented in lung cancer [239]. However, in our study, no significant correlation was found between B7-H3 expression and the density of intratumoral CD8+ T-cell infiltration. CD8+ lymphocytes are key effectors of anti-tumor immune responses regulated by immune checkpoints, and their functional exhaustion represents a major mechanism by which tumors evade immune surveillance [240]. Notably, high B7-H3 expression has been associated with a reduced number of CD8+ cells in osteosarcoma, endometrial cancer, and lung cancer [65,241,242]. In CRC, however, the currently available data remains inconclusive and contradictory.

## 6. Conclusions and Perspectives

In the era of constantly increasing therapeutic options for cancer patients, developing novel effective strategies in response to rapidly occurring drug resistance stands as a main challenge for modern oncology. To achieve this, discovering new immune checkpoint molecules or pathways responsible for therapy resistance is crucial. Thus, B7-H3, studied extensively in recent years, emerges as an attractive target for clinical trials and future treatment strategies [132]. Through numerous signaling pathways, B7-H3 alters the immune and non-immune functions of diverse cell populations in the event of tumorigenesis, facilitating the pathological processes and contributing to disease progression [13,132]. Strategies involving B7-H3 targeting include CAR-T therapies, ADCs, ADCC, mAbs, or radioimmunotherapy and are being evaluated in multiple clinical trials [132]. In preclinical studies, CAR-T therapies against B7-H3 have shown promising results in malignancies such as melanoma, glioblastoma, prostate cancer, and RCC [78,84,86,93,103,153]. ADCs targeting B7-H3 exerted cytotoxic effects on glioblastoma, neuroblastoma cells, prostate cancer, and craniopharyngioma models, while bispecific ADCs, possessing the ability to target PD-L1, activated cell death in laryngeal squamous cell carcinoma cell lines [96,106,154,179]. The combinations of the mentioned strategies could also deliver potent results. At the same time, B7-H3 targeting mAbs could enhance the efficacy of other immunotherapeutic agents by converting immune-cold tumors into immune-hot ones [97,108]. Such combined treatment models could become a direction of future research, delivering crucial data regarding therapeutic options to optimize anti-cancer properties of therapeutic agents.

Despite promising results of preclinical studies, B7-H3 targeting in clinical trials was related to many toxic effects with unclear pathogenesis. Moreover, severe AEs led to the termination of some clinical trials concerning the use of anti-B7-H3 mAbs or radioimmunotherapy [220,223]. Combined therapies could be an answer to this issue. Investigating the effects of RIT and mAbs, or targeting of multiple immune checkpoint molecules, may bring a breakthrough in anti-B7-H3 therapies, allowing for the development of safer, more effective strategies. Nevertheless, several issues, including the nature of the B7-H3 receptor, molecular mechanisms behind AEs, or potential biomarkers for anti-B7-H3 therapy response, remain poorly explored and require clarification. In conclusion, immunotherapies involving B7-H3 present great potential for the treatment of various malignancies, but numerous challenges arising around them must be addressed. Encouraging further research on the topic may help shed more light on the complex problematics of the efficacy and safety of B7-H3 targeting.

## Figures and Tables

**Table 1:** B7-H3 role in shaping the immune landscape in tumors. In most malignancies discussed, B7-H3 upregulation exerts immunosuppressive function, inhibiting T-cell and NK-cell activity, while increasing TAM, Treg, or neutrophil numbers.

Cancer	Influence of B7-H3 Upregulation on the Immune Landscape		References
	Increase in	Decrease in	
Lung cancer	PD-L1^+^ macrophages (lung squamous cell carcinoma)	CD8+ T-cell activity (lung adenocarcinoma)	[48,49]
Breast cancer	-	T-cells/CD8+ cells	[50]
Cervical cancer	-	CD8+ T-cells	[51,52]
Ovarian cancer	M2 macrophages	CD8+ T-cells, NK cells	[22,25,53,54,55]
Prostate cancer	Tregs (B7-H3 mRNA)	Dendritic cells, CD4+ T-cells	[37,56]
Renal cancer	Tregs, TAMs; T-cell exhaustion score	-	[15,57]
Bladder cancer	TAMs	-	[58]
Melanoma	-	CD8+ T-cells, NK cells	[59]
Gliomas	T-cell activation	T-cell anti-tumor activity	[60]
Neuroblastoma	TAMs	NK cells	[45]
Medulloblastoma	-	Tγδ cells, TILs	[61]
Craniopharyngioma	TAMs	TILs	[62]

**Table 2:** Ongoing clinical trials of CAR-T cell therapies targeting B7-H3.

Drug	Immunotherapy Type	Trial	Type of Study	Tumor Types	Status
TH027	CAR T cells	NCT06951425	Early phase I clinical trial	Relapsed/Refractory Solid Tumors	Not yet recruiting
MT027	CAR T cells	NCT06912152	Phase I clinical trial	Advanced Peritoneal Malignancies and Abdominal Metastatic Solid Tumors	Recruiting
Super Hi-TCR-T cells	CAR T cells	NCT06902389	Phase I/II clinical trial	Advanced Hepatocellular Carcinoma	Not yet recruiting
GD2/B7-H3 CAR-T therapy	CAR T cells	NCT06836505	Phase I/II clinical trial	Relapsed/refractory Neuroblastoma and Desmoplastic Small Round Cell Tumors	Recruiting
Allogeneic B7-H3 CAR-γδT-Cell Therapy	CAR T cells	NCT06825455	Early phase I clinical trial	Advanced Solid Tumors	Not yet recruiting
MT027	CAR T cells	NCT06742593	Phase I clinical trial	Brain, Meninges, and Spinal Cord Metastatic Solid Tumors	Not yet recruiting
MT027	CAR T cells	NCT06737146	Phase I clinical trial	Recurrent or Progressive High-grade Glioma	Not yet recruiting
MT027	CAR T cells	NCT06726564	Phase I clinical trial	Pleural Malignant Tumors	Recruiting
WL276 CAR T Cells	CAR T cells	NCT06691308	Early phase I clinical trial	CD276 Positive Recurrent or Progressive Glioblastoma	Not yet recruiting
Autologous B7-H3 CAR T Cells	CAR T cells	NCT06646627	Phase I clinical trial	Recurrent Platinum-resistant Ovarian Tumors	Recruiting
CMD03	CAR T cells	NCT06612645	Phase I clinical trial	Relapse or Refractory Solid Tumors	Recruiting
QH104 Cell Injection	CAR T cells	NCT06592092	Not applicable clinical trial	Meningeal Metastases of B7-H3+ Solid Tumors	Recruiting
Autologous B7-H3 CAR T Cells	CAR T cells	NCT06500819	Phase I clinical trial	Relapsed/Refractory Solid Tumors	Recruiting
Anti-B7-H3 CAR T-cell injection (TX103)	CAR T cells	NCT06482905	Phase I clinical trial	Recurrent or Progressive Grade 4 Glioma	Recruiting
UTAA06 Injection	CAR T cells	NCT06372236	Phase I clinical trial	Advanced Malignant Solid Tumors	Recruiting
iC9-CAR.B7-H3 T Cells	CAR T cells	NCT06347068	Phase I clinical trial	Relapsed/Refractory Triple-Negative Breast Cancer	Recruiting
iC9-CAR.B7-H3 T Cells	CAR T cells	NCT06305299	Phase I clinical trial	Ovarian Cancer	Recruiting
B7-H3 specific CAR T-cell with IL-7Ra signaling domain	CAR T cells	NCT06221553	Phase I clinical trial	Diffuse Intrinsic Pontine Glioma	Recruiting
iC9-CAR.B7-H3 T-cell infusion	CAR T cells	NCT06158139	Phase I clinical trial	Pancreas Cancer, Resistant Cancer	Recruiting
Loc3CAR: Locoregional Delivery of B7-H3-CAR T Cells	CAR T cells	NCT05835687	Phase I clinical trial	Pediatric Patients with Primary CNS Tumors	Recruiting
SC-CAR4BRAIN	CAR T cells	NCT05768880	Phase I clinical trial	Pediatric Diffuse Intrinsic Pontine Glioma, Diffuse Midline Glioma, Recurrent/Refractory Central Nervous System Tumors	Recruiting
Targeted IL-13 Rα2 UCAR T-cell injection, Targeted B7-H3 UCAR T-cell injection	CAR T cells	NCT05752877	Not applicable clinical trial	Advanced Glioma	Recruiting
UTAA06 injection	CAR T cells	NCT05731219	Phase I clinical trial	Relapsed/Refractory Acute Myeloid Leukemia	Recruiting
UTAA06 Injection	CAR T cells	NCT05722171	Phase I clinical trial	Relapsed/Refractory Acute Myeloid Leukemia	Unknown status
TAA06 injection	CAR T cells	NCT05562024	Phase I clinical trial	Relapsed/Refractory Neuroblastoma	Recruiting
KT095 CAR-T injection	CAR T cells	NCT05515185	Early phase I clinical trial	Advanced Gastrointestinal Tumors, Advanced Solid Tumor	Unknown status
B7-H3-CAR T cells	CAR T cells	NCT05474378	Phase I clinical trial	Recurrent Glioblastoma Multiforme (GBM), Brain and Nervous System	Recruiting
B7-H3-CAR T cells	CAR T cells	NCT05366179	Phase I clinical trial	Refractory Glioblastoma	Recruiting
EGFR/B7-H3 CAR-T	CAR T cells	NCT05341492	Early phase I clinical trial	EGFR/ B7-H3-positive Advanced Lung Cancer, EGFR/ B7-H3-Positive Advanced Triple-Negative Breast Cancer	Recruiting
fhB7-H3.CAR-Ts	CAR T cells	NCT05323201	Phase I/II clinical trial	Advanced Liver Cancer	Recruiting
B7-H3-CAR T cells	CAR T cells	NCT05241392	Phase I clinical trial	Recurrent Glioblastoma	Active, not recruiting
fhB7-H3.CAR-Ts	CAR T cells	NCT05211557	Phase I/II clinical trial	Recurrent Malignant Ovarian Cancer	Recruiting
TAA06 injection	CAR T cells	NCT05190185	Phase I clinical trial	Advanced Solid Tumors	Unknown status
CD276 CART -cells	CAR T cells	NCT05143151	Phase I/II clinical trial	Advanced Pancreatic Carcinoma	Recruiting
Autologous B7-H3 CAR T Cells	CAR T cells	NCT04897321	Phase I clinical trial	Pediatric Solid Tumors	Recruiting
TILs and CAR-TILs targeting B7-H3, HER2, Mesothelin, PSCA, MUC1, Lewis-Y, GPC3, AXL, EGFR, Claudin18.2/6, ROR1, GD1	CAR T cells	NCT04842812	Phase I clinical trial	Advanced Solid Tumors	Recruiting
TAA06 injection	CAR T cells	NCT04692948	Not applicable clinical trial	Relapsed/Refractory Acute Myeloid Leukemia	Unknown status
Autologous B7-H3 CAR T Cells	CAR T cells	NCT04670068	Phase I clinical trial	Recurrent Epithelial Ovarian Cancer	Active, not recruiting
CD19CAR-CD3Zeta-4-1BB-Expressing Autologous T-Lymphocyte Cells	CAR T cells	NCT04544592	Phase I/II clinical trial	Elapsed and/or Refractory B-Cell Acute Lymphoblastic Leukemia (B-ALL), B-Cell Non-Hodgkin Lymphoma (B-NHL)	Recruiting
Second-generation 4-1BBζ B7-H3-EGFRt-DHFR	CAR T cells	NCT04483778	Phase I clinical trial	Recurrent/Refractory Solid Tumors in Children and Young Adults	Active, not recruiting
4SCAR-276	CAR T cells	NCT04432649	Phase I/II clinical trial	Solid Tumors	Unknown status
B7-H3-CAR T cells	CAR T cells	NCT04385173	Phase I clinical trial	Recurrent/Refractory Glioblastoma	Recruiting
SCRI-CARB7-H3(s)	CAR T cells	NCT04185038	Phase I clinical trial	Diffuse Intrinsic Pontine Glioma/Diffuse Midline Glioma, Recurrent/Refractory Pediatric Central Nervous System Tumors	Recruiting
B7-H3-CAR T cells	CAR T cells	NCT04077866	Phase I/II clinical trial	Recurrent/Refractory Glioblastoma	Recruiting
CAR T-cells targeting GPC3, Mesothelin, Claudin18.2, GUCY2C, B7-H3, PSCA, PSMA, MUC1, TGFβ, HER2, Lewis-Y, AXL, or EGFR	CAR T cells	NCT03198052	Phase I clinical trial	Advanced cancer that expresses GPC3/Mesothelin/Claudin18.2/GUCY2C/B7-H3/PSCA/PSMA/MUC1/TGFβ/HER2/Lewis-Y/AXL/EGFR protein	Recruiting
Antigen-specific IgT cells	CAR T cells	NCT03170141	Phase I clinical trial	Glioblastoma Multiforme	Enrolling by invitation

**Table 3:** Ongoing clinical trials of ADC therapies targeting B7-H3.

Drug	Immunotherapy Type	Trial	Type of Study	Tumor Types	Status
MHB088C	B7-H3 ADC	NCT06951243	Phase II clinical trial	Extrapulmonary Neuroendocrine Cancer	Not yet recruiting
HS-20093	B7-H3 ADC	NCT06825624	Phase I clinical trial	Advanced Metastatic Colorectal Cancer	Recruiting
HS-20093	B7-H3 ADC	NCT06699576	Phase Ib clinical trial	Bone and Soft Tissue Sarcoma	Not yet recruiting
YL201	B7-H3 ADC	NCT06629597	Phase III clinical trial	Nasopharyngeal Carcinoma	Recruiting
HS-20117	B7-H3 ADC	NCT06621563	Phase I clinical trial	Advanced Solid Tumors	Recruiting
YL201	B7-H3 ADC	NCT06612151	Phase III clinical trial	Relapsed Small Cell Lung Cancer	Recruiting
BGB-C354	B7-H3 ADC	NCT06422520	Phase I clinical trial	Advanced Solid Tumors	Recruiting
YL201	B7-H3 ADC	NCT06394414	Phase I clinical trial	Advanced Solid Tumors	Recruiting
I-DXd in Combination with Atezolizumab with or without Carboplatin	B7-H3 ADC	NCT06362252	Phase I/II clinical trial	Extensive Stage-Small Cell Lung Cancer	Recruiting
MGC026	B7-H3 ADC	NCT06242470	Phase I clinical trial	Advanced Solid Tumors	Recruiting
YL201	B7-H3 ADC	NCT06241846	Phase II clinical trial	Metastatic Castration-resistant Prostate Cancer	Recruiting
Ifinatamab Deruxtecan (I-DXd)	B7-H3 ADC	NCT06203210	Phase III clinical trial	Relapsed Small Cell Lung Cancer (SCLC)	Recruiting
HS-20093	B7-H3 ADC	NCT06112704	Phase II clinical trial	Advanced Esophageal Carcinoma, Advanced Solid Tumors	Recruiting
HS-20093	B7-H3 ADC	NCT06052423	Phase II clinical trial	Extensive Stage Small Cell Lung Cancer (ES-SCLC)	Not yet recruiting
IBB0979	B7-H3-IL10 immunocytokine	NCT05991583	Phase I/II clinical trial	Advanced Malignant Tumors	Recruiting
DB-1311/BNT324	B7-H3 ADC	NCT05914116	Phase I/IIa clinical trial	Advanced/Metastatic Solid Tumors	Recruiting
Vobramitamab duocarmazine (MGC018)/Lorigerlimab (MGD019)	B7-H3 ADC/Bispecific DART^®^ Protein Binding PD-1 and CTLA-4	NCT05293496	Phase I/Ib clinical trial	Relapsed/Refractory, Unresectable, Locally Advanced/Metastatic Solid Tumors	Active, not recruiting
Ifinatamab Deruxtecan (I-DXd)	B7-H3 ADC	NCT05280470	Phase II clinical trial	Extensive-stage Small Cell Lung Cancer (ES-SCLC)	Active, not recruiting
HS-20093	B7-H3 ADC	NCT05276609	Phase I clinical trial	Advanced Solid Tumors	Unknown status
BNT116	B7-H3 ADC	NCT05142189	Phase I clinical trial	Advanced Non-small Cell Lung Cancer	Recruiting
Ifinatamab deruxtecan (I-DXd)	B7-H3 ADC	NCT04145622	Phase I/II clinical trial	Advanced Solid Malignant Tumors	Recruiting

**Table 4:** Ongoing clinical trials of radioimmunotherapies targeting B7-H3.

Drug	Immunotherapy Type	Trial	Type of Study	Tumor Types	Status
131 I-omburtamab	Radioimmunotherapy	NCT04022213	Phase II clinical trial	Desmoplastic Small Round Cell Tumor	Active, not recruiting
131 I-omburtamab	Radioimmunotherapy	NCT04743661	Phase II clinical trial	Recurrent Medulloblastoma, Recurrent Ependymoma	Active, not recruiting
131 I-omburtamab	Radioimmunotherapy	NCT05064306	Expanded access	Central Nervous System/Leptomeningeal Neoplasms in Children and Young Adults	Available

**Table 5:** Completed clinical trials targeting B7-H3.

Trial	Type of Study	Study Design	Enrollment	Drug	Immunotherapy Type	Tumor Types	Conclusion
NCT02628535	Phase I clinical trial	Open-label, dose escalation, cohort expansion, efficacy follow-up study	67	MGD009	B7-H3- CD3 dual-affinity re-targeting (DART) protein	Advanced Solid Tumors	Study terminated—Business decision (not for safety reasons) AE:NA
NCT02982941	Phase I clinical trial	Open-label, dose escalation, cohort expansion, and efficacy follow-up study	25	Enoblituzumab (MGA271)	IgG1κ B7-H3 monoclonal antibody, anti-B7-H3 ADCC	Pediatric Solid Tumors	Study completed AE:NA
NCT03406949	Phase I clinical trial	Open-label, dose escalation, and cohort expansion study	25	MGD009/MGA012	B7-H3- CD3 dual-affinity re-targeting (DART) protein with anti-PD-1 Antibody	Advanced Solid Tumors	Study completed AE:NA
NCT02381314	Phase I clinical trial	Open-label, dose escalation, and cohort expansion study	24	Enoblituzumab (MGA271)/ Ipilimumab	IgG1κ B7-H3 monoclonal antibody, Anti-B7-H3 ADCC/CTLA-4 monoclonal antibody	Advanced Solid Tumors	Study completed AE:NA
NCT03729596	Phase I/II clinical trial	Open-label, dose-escalation study	143	Vobramitamab duocarmazine (MGC018) with or without (MGA012)	Anti- B7-H3 ADC alone and in combination with anti-PD-1 antibody	Advanced Solid Tumors	Study terminated—Business decision (not for safety AE: In ≥10% of patients, neutropenia asthenia, palmar–plantar erythrodysesthesia, headache.
NCT04634825	Phase II clinical trial	Open-label study	62	Enoblituzumab (MGA271) plus Retifanlimab (MGA012)/ Tebotelimab (MGD013)	Anti-B7-H3 ADCC plus Anti-PD-1 antibody/PD-1 X LAG-3 bispecific DART molecule	Head and Neck Squamous Cell Carcinoma	Study terminated—Safety concerns. AE: Retifanlimab cohort, In ≥10% of patients with hypothyroidism, fatigue, infusion-related reaction, and increased lipase level. Tebotelimab cohort, asthenia, infusion related reaction, increased N-terminal prohormone brain natriuretic peptide level, and headache.
NCT04630769	Phase I clinical trial	Open-label study	3	Intraperitoneal FATE FT516 and Interleukin-2 (IL-2) with Enoblituzumab (MGA271)	Allogeneic natural killer (NK) cells, expressing CD16 (FT516) and IL-2 with Fc-optimized monoclonal antibody targeting B7-H3	Ovarian, Fallopian Tube, and Primary Peritoneal Cancer	Study completed. AE: nausea, headache, abdominal pain, anemia, fatigue, decreased lymphocyte count, decreased white blood cell count, anorexia, and hypoalbuminemia.
NCT02192567	Phase I clinical trial	Open-label, sequential dose escalation and expansion study	19	DS-5573a	Anti- B7-H3 ADC	Advanced Solid Tumors	Study terminated —Business decision (not for safety reasons) AE:NA
NCT01391143	Phase I clinical trial	Open-label, multi-dose, single-arm, multi-center, dose-escalation study	179	Enoblituzumab (MGA271)	IgG1κ B7-H3 monoclonal antibody, Anti-B7-H3 ADCC	Solid Tumors	Study completed. Disease stabilization and tumor shrinkage (ranging from 2% to 69%) across various tumor types. MGA271 demonstrated good tolerance, with no dose-limiting toxicity. AE: Grade 3/4 E was observed in 6% of patients.
NCT02982941	Phase I clinical trial	Open-label, dose escalation and cohort expansion study	25	Enoblituzumab (MGA271)	IgG1κ B7-H3 monoclonal antibody, Anti-B7-H3 ADCC	NB, sarcomas	Study completed. Results: NA AE: NA
NCT02475213	Phase I clinical trial	Open-label, dose escalation study	145	Enoblituzumab (MGA271) in combination with Pembrolizumab or MGA012	IgG1κ B7-H3 monoclonal antibody, anti-PD-1 antibody	Melanoma, HNSCC, NSCLCC, Urothelial Carcinoma	Study completed. Metastatic NSCLC 36% ORR, recurrent/metastatic HNSCC 33% ORR AE: acceptable safety profile.
NCT01502917	Phase I clinical trial	Open-label, dose escalation, single-group assignment study	50	Radioactive iodine-labeled monoclonal antibody—124I-Omburtamab	Radioimmunotherapy	Central Nervous System Tumors	Study completed. AE: hemiparesis, dysarthria, ataxia, dysphagia, muscular weakness.
NCT00089245	Phase I clinical trial	Open-label, single-arm study	177	Iodine I 131 MOAB 8H9	Radioimmunotherapy	Central Nervous System Tumors	Study terminated. Median PFS 7.5 years. AE: headache, vomiting, fever, and biochemical abnormalities.
NCT01099644	Phase I clinical trial	Open-label, dose escalation, single-group assignment study	54	131I-omburtamab (131 I-8H9)	Radioimmunotherapy	Solid Tumors Involving the Peritoneum	Study terminated. AE: NA
NCT03275402	Phase II/III clinical trial	Open-label, multicenter, single-group assignment study	52	131I-omburtamab (131 I-8H9)	Murine IgG1 monoclonal antibody radiolabeled with iodine-131, radioimmunotherapy	Neuroblastoma Central Nervous System/Leptomeningeal Metastases	Study terminated. 3-year OS 0.65 (0.49 to 0.78). AE: lymphopenia, intracranial hemorrhage, and decreased platelet count.

## Data Availability

No new data were created or analyzed in this study. Data sharing is not applicable to this article.
